# A Novel Admixture-Based Pharmacogenetic Approach to Refine Warfarin Dosing in Caribbean Hispanics

**DOI:** 10.1371/journal.pone.0145480

**Published:** 2016-01-08

**Authors:** Jorge Duconge, Alga S. Ramos, Karla Claudio-Campos, Giselle Rivera-Miranda, Luis Bermúdez-Bosch, Jessicca Y. Renta, Carmen L. Cadilla, Iadelisse Cruz, Juan F. Feliu, Cunegundo Vergara, Gualberto Ruaño

**Affiliations:** 1 Pharmaceutical Sciences Department, School of Pharmacy, University of Puerto Rico Medical Sciences Campus (UPR-MSC), San Juan, Puerto Rico, United States of America; 2 Department of Pharmacology and Toxicology, School of Medicine, University of Puerto Rico Medical Sciences Campus (UPR-MSC), San Juan, Puerto Rico, United States of America; 3 Pharmacy Service, VA Caribbean Healthcare Systems (VACHS), San Juan, Puerto Rico, United States of America; 4 Molecular Genetics Lab, Department of Biochemistry, School of Medicine, University of Puerto Rico Medical Sciences Campus (UPR-MSC), San Juan, Puerto Rico, United States of America; 5 Brownstone Outpatient Clinic, Hartford Hospital, Hartford, CT, United States of America; 6 Genomas Inc., Hartford, CT, United States of America; Institute of Bioengineering and Nanotechnology, SINGAPORE

## Abstract

**Aim:**

This study is aimed at developing a novel admixture-adjusted pharmacogenomic approach to individually refine warfarin dosing in Caribbean Hispanic patients.

**Patients & Methods:**

A multiple linear regression analysis of effective warfarin doses versus relevant genotypes, admixture, clinical and demographic factors was performed in 255 patients and further validated externally in another cohort of 55 individuals.

**Results:**

The admixture-adjusted, genotype-guided warfarin dosing refinement algorithm developed in Caribbean Hispanics showed better predictability (R^2^ = 0.70, MAE = 0.72mg/day) than a clinical algorithm that excluded genotypes and admixture (R^2^ = 0.60, MAE = 0.99mg/day), and outperformed two prior pharmacogenetic algorithms in predicting effective dose in this population. For patients at the highest risk of adverse events, 45.5% of the dose predictions using the developed pharmacogenetic model resulted in ideal dose as compared with only 29% when using the clinical non-genetic algorithm (p<0.001). The admixture-driven pharmacogenetic algorithm predicted 58% of warfarin dose variance when externally validated in 55 individuals from an independent validation cohort (MAE = 0.89 mg/day, 24% mean bias).

**Conclusions:**

Results supported our rationale to incorporate individual’s genotypes and unique admixture metrics into pharmacogenetic refinement models in order to increase predictability when expanding them to admixed populations like Caribbean Hispanics.

**Trial Registration:**

ClinicalTrials.gov NCT01318057

## Introduction

Warfarin is an oral anticoagulant used to treat or prevent multiple thromboembolic disorders, including atrial fibrillation, heart valve replacement, recurrent stroke, deep vein thrombosis, pulmonary embolism, acute myocardial infarction and cerebrovascular accidents [1–2]. Although several clinical trials have compared its effectiveness and safety to the new oral anticoagulants (NOACs) [3], warfarin continues to be the standard of care in oral anticoagulation. According to IMS Health, over 19 million prescriptions of warfarin sodium were dispensed annually in the US by 2012, ranking #18^th^ overall [4–5]. However, it is still in the top 10 drug products by reports of adverse events in outpatients [6–7]. Despite its proven clinical benefits and its use for a long time, warfarin therapy is still a challenge due to a wide inter-individual variation in dose requirements, narrow therapeutic range and risk of serious bleeding [1,8].

Pharmacogenetic-guided warfarin dosing algorithms have been developed to explain about 60% of dose variability in various populations, mostly in Caucasians [1]. Nonetheless, their impact on clinical outcomes remains controversial and reimbursement for genotyping is debated [9–10]. On the other hand, Hispanics have been largely excluded from the corresponding derivation cohorts [11–13]. Accordingly, these algorithms are limited to account for the effect of multi-hybrid admixtures and the unique stratification observed in Hispanics, which increase dramatically the disparities in translating benefits from pharmacogenomics to this medically underserved, minority population. We believe that a better approach for global pharmacogenetics (PGt) is to guide warfarin dosing by using a pharmacogenetic-based algorithm that also accounts for the effect of admixture or ancestry proportions.

Fostered by an urgent need to eliminate potential ethnic disparities in health care while implementing the personalized medicine paradigm, this study is aimed at developing a novel admixture-adjusted pharmacogenomic approach to predict individual maintenance doses of warfarin, after the fourth day of therapy (refinement model), in Caribbean Hispanic patients receiving anticoagulation therapy at the Veterans Affairs Caribbean Healthcare System (VACHS). We also compare the predictability of the developed algorithm in Caribbean Hispanics versus a clinical non-genetic algorithm and two other previously established pharmacogenetic-guided warfarin dosing models.

## Methods

### Study Design and Patient Cohort

All research activities involving human participants in this study were properly approved by the Institutional Review Boards (IRBs) at both VACHS (#00558) and UPR-MSC (A4070109) in November 2010 (S1 Text). All clinical investigations were conducted according to the principles expressed in the Declaration of Helsinki. Written informed consent was obtained from each single participant of the study prior to enrollment (S2 Text and S3 Text).

**Fig 1:**
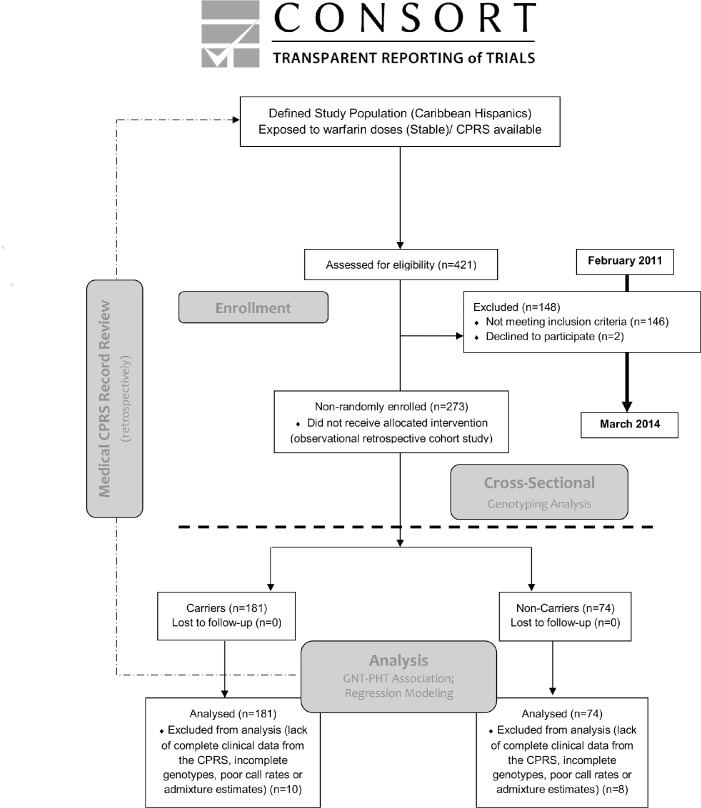
CONSORT flowchart illustrating an open-label, single-center, population-based, observational, retrospective cohorts study with cross-sectional genotyping analysis. The study uses existing data collected in the past (CPRS) to preliminarily identify eligible participants from the defined study population (i.e., Caribbean Hispanics), determine their stabilization status and retrieve relevant covariates for performing regression analysis and association testing. GNT stands for genotypes. PHT stands for phenotypes (e.g., therapeutic warfarin dose). CPRS stands for computerized patient record system.

This is an open-label, single-center, population-based, observational, retrospective cohort study (ClinicalTrial.gov Identifier NCT01318057) as depicted in Fig 1. Four hundred and twenty one (N = 421) warfarin-treated outpatients from the VACHS anticoagulation clinics in San Juan, Puerto Rico, were assessed for eligibility between February 2011 and March 2014. A total of 275 eligible patients who met inclusion criteria were approached regarding participation in this trial. Two patients declined to participate in the study, resulting in 273 subjects enrolled between March 22 and April 7, 2011. To be in compliance with the Ethical Principles for Medical Research Involving Human Subjects, the first participant was recruited 5 days after the study registration date in ClinicalTrials.gov (March 17, 2011). Another eighteen (18) patients were excluded from further analysis due to lack of complete clinical data from the CPRS and incomplete genotype information (e.g., poor call rates) or admixture estimates. This resulted in 255 patients available to include their clinical, demographic and genomic data for full analysis. Because this study required a blood sample at enrollment followed by a retrospective CPRS medical record review, no patients were withdrawn or lost to follow-up. The study completion date was July 2014. The authors confirm that all ongoing and related trials for this drug/intervention are registered.

The eligible candidates were Caribbean Hispanics from Puerto Rico (≥21 years-old). The anticoagulation clinic at this urban medical institution serves a population of over 1,640 patients, of which approximately 95% are Caribbean Hispanics who reside in the Commonwealth of Puerto Rico. The subjects who met the inclusion criteria–stable warfarin dose with at least three consecutive INR measurements on target (2–3 or 2.5–3.5, according to indication for warfarin use) for the same average weekly dose, no anemia, malignancies, renal failure, hepatic disease or any other acute illness, no use of other anticoagulants–were selected from the study population based on their warfarin initiation date (after January 2001). The requirement for warfarin therapy was determined on the basis of the American College of Chest Physicians (ACCP) evidence-based clinical practice guidelines [14–15]. Patients with low compliance (<80% medication possession ratio) were excluded. Although varying from case to case, the majority of patients were initiated on warfarin therapy following induction regimens that rely on age-adjusted fixed-dose schemes.

### Genetic Assays

The participants provided a small blood sample (5ml) for cross-sectional genotyping as well as to infer genetic structure and measure admixture. Whole blood samples were processed for genomic DNA extraction using a QIAamp DNA Blood Maxi Kit (spin protocol; QIAGEN Inc., Venlo, Limburg) according to manufacturer’s instructions. DNA quantification was performed by using the NanoDrop® Spectrophotometer (ThermoScientific, Wilmington, DE, US) and fluorescent staining of double-stranded DNA (PicoGreen® dsDNA Quantitation Kit, Molecular Probes, Eugene, OR, US). Fluorescent intensity was measured using a fluorescent micro-titer plate reader (BMG Labtech, FluoStar Optima, Ortenberg, Germany). Isolated DNA specimens were first tested for relevant polymorphisms on candidate warfarin-related pharmacogenes using the DMET^TM^ plus assay (Affymetrix®, Santa Clara, CA, USA) at the RCMI Core Lab of Molecular Genetics (UPR-MSC), and confirmed by either pre-developed TaqMan® Genotyping Assay Reagent kits for Allelic Discrimination (Applied Biosystems, CA, USA) or Tag-It™ Mutation Detection Assay for warfarin (LUMINEX xMAP® Technology) at the CLIA-certified Laboratory of Personalized Health (Genomas Inc., Hartford, CT). The African-related rare variant CYP2C9*8 (c.449G>A; p.R150H) and two other polymorphisms in the promoter region of the gene (i.e., c.-1188T>C and c.-1766T>C), which are not included in the commercial DMET^TM^ plus panel, were individually detected by their corresponding TaqMan® SNP Genotyping Custom Assay Reagent kits for Allelic Discrimination (Life Technologies, Carlsbad, CA, USA) following manufacturer’s instructions. A full description of these assays and a complete list of variants ascertained can be found elsewhere [16–21]. The *CYP2C9* allele designations refer to those defined by the Cytochrome P450 Allele Nomenclature Committee [22–23].

Samples were also genotyped on a *PhysioGenomic* (PG) array that interrogates 384 single nucleotide polymorphisms (SNPs) and ancestry informative markers across 222 cardio-metabolic and neuroendocrine genes [24]. Careful manual analysis was performed on the alignments underlying the genotype calls using GenCall 6.1.3.24 and 50 SNPs with a slight degree of uncertainty about calling accuracy were not included in the analysis, leaving 332 SNPs from 196 genes. Genotyping was accomplished using Illumina® BeadArray™ technology [25–26]. A full list of the genes and SNPs in the PG chip is provided in our physiogenomic analysis study [24].

Departure from Hardy-Weinberg equilibrium (HWE) was estimated under the null hypothesis of the predictable segregation ratio of specific matching genotypes (p>0.05) by use of χ2 goodness of fit test with 1 degree of freedom. Clinical non-genetic and demographic information from the participants was retrospectively extracted from the corresponding computerized patient record system (CPRS). Individual warfarin doses at the first day of therapy (D1, usually 2.5 or 5 mg as per the clinical nomogram in the VACHS anticoagulation clinic) were retrieved from the corresponding records in CPRS. Statistical calculation indicated 80% power to detect SNPs with allele frequencies of 0.1–0.4 in the study sample, accounting for 10% of phenotypic variation at <5% significance level.

### Quantification of Admixture

A Bayesian’s clustering of individuals according to their maximal PG-derived genotype sharing was performed using the *STRUCTURE* v2.3.4 software package [27–29]. Three Markov Chain Monte Carlo (MCMC) runs were performed using default parameters and an assumed cluster size (K) of 3, which was chosen based on our knowledge of migration history in the Caribbean basin to reflect admixture from Amerindian (Tainos Arawaks), West-African, and European ancestors as well as to match previous reports for a 3-hybrid model [30–35]. A burn-in time of 30,000 iterations was followed by 70,000 further iterations. The log likelihood for each run was determined as *a posteriori* probability for the different numbers of clusters. Each individual was represented by a dot in the triangle plot of ancestral proportions. Proximity values (between 0 and 1) corresponding to each of 3 vertices in the triangle plot were calculated for each dot. A categorical criterion of proximity was added requiring that in order for an individual to be assigned to a specific cluster, its higher proximity value must be greater than the other two by at least 0.2. This criterion excluded those individuals not clearly positioned towards any vertex but rather located near the middle of the triangle (i.e., the mixed ones).

### Multiple Linear Regression Modeling

We selected all the patients with complete genetic and clinical data as the “derivation cohort” (n = 255) for developing dose-prediction models. Like in earlier reports, this “derivation cohort” size allowed inclusion of up to 13 independent variables in the final model [36]. A sample of another 55 patients from the Brownstone outpatient clinic in Hartford Hospital (Hartford, CT, USA) constituted the external ‘validation cohort’, which was used for testing validity of the developed pharmacogenetic model. Patients in the validation cohort were selected based on the same inclusion criteria as those in the derivation cohort. The investigators who performed the modeling and analysis did not have access to this validation set until after the final model was selected.

A forward stepwise entry and backward stepwise elimination approach in multiple linear regression analysis was used to ascertain the association between average therapeutic warfarin doses (dependent variable) and individual's genetics (i.e., genotypes and admixture index), demographics (i.e., age, body size) and non-genetic clinical information (i.e., dose-adjusted INR, target INR, co-meds use, indications, smoking status, comorbidities, diet) available on day 3 of warfarin therapy. Clinically relevant genetic variants on the following candidate pharmacogenes were tested: *CYP2C9*, *VKORC1*, *EPHX1*, *CYP4F2*, and *NQO1*. Variables were included in the final linear regression model if they were significantly (p<0.05) associated with the daily warfarin dose or were marginally significant (0.05 ≤ p ≤ 0.20) with strong biological plausibility.

The average therapeutic warfarin dose (mg/day) is defined as the dose that led to stable therapeutic anticoagulation levels (i.e., INR values in range on at least three consecutive visits for the same average dose). The numerical designations of “0” for the CYP2C9*1/*1 genotype (wild-type); “1” for the *1/*n genotypes (n stands for *3,*5,*6,*8,*11 “loss-of-function” variants detected in the genotyping assay) and “2” for *n/*n genotypes (e.g., *2/*2, *3/*3, *3/*5) were used to correspond to the relative capacity of the CYP system to metabolize warfarin. *VKORC1*-1639 AA status is a carrier code, where 1 = AA and 0 = otherwise; *VKORC1*-1639 GA status is another code, where 1 = GA and 0 = otherwise. Likewise, the numerical designations of “0” was used for the wild-types on either *CYP4F2* or *NQO1* loci; whereas, “1” was set for the respective single carrier (i.e., CYP4F2*1/*3 or NQO1*1/*2) and “2” for the double-carriers of the corresponding allele variants in each of these two warfarin-related genetic loci (i.e., CYP4F2*3/*3 or NQO1*2/*2). The admixture index was defined as follows (1 = European ancestry, 2 = Native American ancestry, 3 = African ancestry, 4 = Admixed individual). All discrete variables were treated as dichotomous (e.g., Amiodarone user: Yes = 1, No = 0). Effect on the estimates of the maintenance warfarin dose was calculated per number of variant alleles (genetics), per decade (age) and per 0.25-unit increase in the dose-adjusted INR response at day 3.

All discrete covariates entered the analysis transformed as dummy variables to quantify how the individual categories affected daily mean warfarin dose. To this purpose, an inter-correlation matrix to identify genetic, demographic, and other clinical variables that correlate with maintenance warfarin daily dose was constructed.

Comparisons of the arithmetic mean values (or percentages) between carriers and wild-types were calculated with the use of the two-tailed Mann–Whitney U/Wilcoxon’s rank-sum test (for warfarin dose, age, height, weight, target INR range, INR/dose at third day) and χ2 or Fisher’s exact test (for co-medications, smoking status, race and comorbidities) at 5% significance. Analyses of variance to accommodate unequal cell sizes were used to test the effect of genotypes as an independent variable.

#### Model Diagnostics

The mean absolute error (MAE, mg/day, defined as the mean of the absolute values for the difference between the predicted and actual doses) was used to evaluate the model’s predictive accuracy. We selected the final model as the one that had the lowest predictive MAE. The bias of the dosing algorithm estimates (precision) was assessed by calculating the mean percentage of difference from the observed dose, where mean percentage of difference is equal to the MAE between predicted and actual dose divided by the actual dose ([predicted dose—observed dose]/observed dose) × 100%. Finally, the effect size of each independent predictor covariate on the daily dose of warfarin was also computed.

We compared dose predictions from our admixture-adjusted pharmacogenetic model for dose-refinement with those from three other separate models: 1) a clinical algorithm that uses the same equation of the admixture-adjusted pharmacogenetic model, but excluding admixture and genotypes; 2) the Lenzini *et al*. refinement model [36] and 3) the multi-ethnic IWPC algorithm [37]. We evaluated the potential clinical value of each algorithm by calculating the percentage of patients in both study cohorts whose predicted dose of warfarin was within 20% of the actual stable therapeutic dose. In addition, we calculated the percentage of patients for whom the predicted dose according to each algorithm was at least 20% higher than the actual dose (overestimation) or at least 20% lower than the actual dose (underestimation). These values represent a difference of 1 mg/day relative to the traditional starting dose of 5 mg/day, a difference clinicians would define as clinically relevant. We also assessed the performance of the algorithms in three dose groups: participants requiring a low dose (≤3 mg/day), those requiring a high dose (≥7 mg/day) and those requiring intermediate doses (>3 and <7 mg/day) for effective anticoagulation. McNemar’s test of paired proportions was used for the comparisons. Significance level of all statistical analyses was set at p<0.05. Statistical analyses were performed using the SmallSTATA® software v.12.

## Results

### Derivation Cohort

The derivation cohort (n = 255 patients) consists of patients with a mean age of 68 ± 9.81 years, 99% were male and 88% were self-identified in their electronic medical record as Hispanic Whites (Table 1). The average therapeutic warfarin daily dose ranged from 1.43 to 11.07 mg/day, a difference representing approximately five standard deviations. From the total of patients enrolled, 49 patients were sensitive (≤3 mg/day) and 28 were resistant (≥7 mg/day) to warfarin. Of these, 10 patients showed discrepancies between actual dose requirements and dose predictions using a previously developed pharmacogenetic algorithm in Puerto Ricans [38]. Table 1 summarizes the most important facts on demographics and non-genetic clinical data of this study cohort. Atrial fibrillation (AFib, n = 136 patients), deep vein thrombosis (DVT, n = 36 patients) and pulmonary thromboembolism (PE, n = 10) were the most common indications for oral anticoagulant therapy with warfarin in this cohort. Potential drug interactions with warfarin were found with statins (simvastatin and fluvastatin, n = 145) and less common with amiodarone (n = 6). Demographic and clinical variables were similarly distributed between carriers and non-carriers.

**Table 1:** Descriptive clinical (non-genetic) and demographic characteristics of the derivation cohort (N = 255 warfarin-treated patients from VACHS) and the independent sample of 55 warfarin-treated Puerto Ricans from the Brownstone Clinic in Hartford, CT (validation cohort).

Characteristics	Derivation Cohort All Patients (N = 255)	Derivation Cohort Non-carriers (n = 74; %)	Derivation Cohort Carriers (n = 181; %)	*p-*value^a^	Validation Cohort (N = 55)
**Stable Warfarin Dose (mg/day)**^**b**^					
Mean^c^ (±SD)	4.62 (1.77)	5.34 (1.70)	4.33 (1.71)	0.0001	5.0 (2)
Range (min–max)	1.43–11.07	1.57–10.00	1.43–11.07		1.5–10.0
**Age (years)**					
Mean (±SD)	68.1 (10)	68 (10)	68 (10)	0.99	60.7 (14.4)
Range (min–max)	31–94	37–94	31–90		23–90
**Weight (lbs.)**					
Mean (±SD)	186.9 (37)	190 (36)	185 (37)	0.44	179.0 (41.9)
Range (min–max)	87–366	136–366	87–341		114.8–277
**Height (inches)**					
Mean (±SD)	67.8 (7)	67 (2)	67 (3)	0.05	62.7 (3.6)
Range (min–max)	61–76	63–73	61–76		53–69
**Atrial fibrillation**^**d**^**/flutter, *N*(%)**	136 (53)	45 (60.81)	135 (74.59)	0.03	33 (60)
**Deep Vein Thrombosis, *N (%)***	36 (14)	15 (20.27)	28 (15.47)	0.36	10 (18.2)
**Pulmonary embolism, *N* (%)**	10 (4)	6 (8.11)	7 (3.87)	0.21	8 (14.5)
**Other indication, *N* (%)**	73 (28.6)	24 (32.43)	61 (33.70)	0.88	4 (7.3)
**Smoker, *N* (%)**	13 (5.10)	2 (2.70)	11 (6.08)	0.36	N/A
**Amiodarone users, *N* (%)**	6 (2.35)	2 (2.70)	4 (2.21)	0.99	2 (3.6)
**Azoles users, *N* (%)**	1 (0.39)	1 (1.35)	0 (0.00)	0.29	N/A
**Statins users, *N* (%)**	145 (56.86)	38 (51.35)	107 (59.12)	0.27	N/A
**Race**^**e**^					
Whites, *N* (%)	226 (88.63)	61 (82.43)	165 (91.16)	0.05	51 (92.7)
Blacks, *N* (%)	29 (11.37)	13 (17.57)	16 (8.84)		4 (7.3)
**INR/Dose at 3**^**r**^^**d**^ **day**					
Mean (±SD)	0.63 (0.37)	0.57 (0.41)	0.66 (0.36)	0.08	0.65 (0.53)
Range (min-max)	0.14–2.27	0.14–2.26	0.17–2.27		0.14–2.47
**Target INR**					
Mean (±SD)	2.53 (0.13)	2.55 (0.16)	2.52 (0.12)	0.10	2.5 (0.5)
Range (min-max)	2.25–3.00	2.50–3.00	2.25–3.00		2–3.5
**Gender**					
Male, *N* (%)	254 (99.6)	74 (100)	180 (99.45)	0.99	20 (36)
Females, *N* (%)	1 (0.4)	0 (0)	1 (0.55)		35 (64)

^a^*p*-values for the difference between the carriers and non-carriers were calculated with the use of the two-tailed U Mann-Whitney/Wilcoxon’s rank-sum test (for warfarin dose, age, height, weight, target INR, INR/Dose at 3^rd^ day) and chi-square or Fisher’s exact test (for co-medications, smoking status, race, gender and comorbidities). Co-medications (brand name in parenthesis when used): Amiodarone; Statins: Simvastatin, Rosuvastatin (Crestor), Fluvastatin (Lescol) and Pravastatin; Azoles: Fluconazole (Diflucan), Itraconazole, Ketoconazole.

^b^Dose titrated empirically based on current clinical guidelines for warfarin patient management and nomogram at VACHS.

^c^Means refer to “arithmetic” means.

^d^Chronic, paroxysmal or post-operative included. N/A stands for information not available.

^e^One subject self-identified as mestizo; another one declined to self-report his race/ethnicity. Age, height and weight are determined at the time of stabilization. NS means not significant differences (*p≥0*.*05*).

The *CYP2C9* and *VKORC1* polymorphic alleles were highly prevalent in this study population (**Table 2**). Ninety-four (94, 37%) individuals are wild-types for both the *CYP2C9* and the *VKORC1* loci combined; whereas, 161 (63%) presented a variant genotype that are subdivided into 37% single, 21% double, 5% triple and 0.4% quadruple carriers of polymorphic alleles in *CYP2C9* and/or *VKORC1* genes. Among all these patients, 30% had at least one polymorphism in *CYP2C9*, whereas ~62% were carriers of the *VKORC1-*1639G>A promoter variant. All alleles were in Hardy-Weinberg equilibrium.

**Table 2:** Allele and genotype frequency distributions of *CYP2C9*, *CYP4F2*, *NQO1* and *VKORC1* polymorphisms in Hispanic patients treated with warfarin at the VACHS-San Juan, PR (n = 255). Data expressed as percent (Wald-adjusted 95% Confidence Interval, CI). *χ*^2^ <3.84 meet HWE for genotypes, one degree of freedom, p<0.05.

Genotype/Alleles ^a^	n; % (95% CI; Margin of Error)	*χ*^2^ test
***VKORC1*-1639 G>A genotype**		0.41
GG	98; 38.4% (32.7–44.5; 0.06)	
GA	124; 48.6% (42.5–54.7; 0.06)	
AA	33; 13% (9.3–17.6; 0.04)	
G	320; 63% (58.5–66.8; 0.04)	
A	190; 37% (33.2–41.5; 0.04)	
***VKORC1* (c.134T>C, p.V45A, rs104894540): Resistant**		<0.001
TT	254; 99.6% (97.6–99.9; 0.0129)	
TC	1; 0.39% (0.01–2.42; 0.0129)	
T	509; 99.8% (98.8–99.9; 0.0065)	
C	1; 0.2% (0.01–1.22; 0.0065)	
***CYP2C9* SNPs**		3.74
*1/*1	179; 70% (64.3–75.5; 0.05)	
*1/*2	36; 14.1% (10.3–18.9; 0.04)	
*1/*3	17; 6.7% (4.1–10.5; 0.03)	
*1/*4	4; 1.6% (0.5–4.1; 0.02)	
*1/*5	2; 0.8% (0.03–3; 0.01)	
*1/*6	1; 0.4% (0.01–2.5; 0.01)	
*1/*8	3; 1.2% (0.25–3.5; 0.01)	
*1/*11	1; 0.4% (0.01–2.5; 0.01)	
*2/*2	2; 0.8% (0.03–3; 0.01)	
*2/*3	6; 2.4% (0.9–5.2; 0.02)	
*2/*5	1; 0.4% (0.01–2.5; 0.01)	
*3/*5	2; 0.8% (0.03–3; 0.01)	
*3/*6	1; 0.4% (0.01–2.5; 0.01)	
*1	422; 83% (79.2–85.8; 0.03)	
*2	47; 9% (7–12; 0.02)	
*3	26; 5% (3.5–7.4; 0.02)	
*4	4; 0.8% (0.2–2.1; 0.01)	
*5	5; 1% (0.35–2.3; 0.01)	
*6	2; 0.4% (0.01–1.5; 0.01)	
*8	3; 0.6% (0.1–1.8; 0.01)	
*11	1; 0.2% (0.009–1.2; 0.006)	
***CYP2C9* (c.-1188T>C)**^**b**^		1.18
T/T	37; 44% (33.9–54.7; 0.104)	
C/T	34; 40.5% (30.6–51.2; 0.103)	
C/C	13; 15.5% (9.13–24.8; 0.08)	
T	108; 64.3% (56.8–71.1; 0.07)	
C	60; 35.7% (28.9–43.2; 0.07)	
CYP4F2*3 (g.18000G>A, p.V433M, rs2108622)		2.82
*1/*1 (G/G)	203; 79.6% (74.2–84.1; 0.05)	
*1/*3 (G/A)	46; 18% (13.8–23.2; 0.05)	
*3/*3 (A/A)	6; 2.4% (1–5; 0.02)	
*1 (G)	452; 88.5% (85.6–91.1; 0.03)	
*3 (A)	58; 11.5% (8.9–14.4; 0.03)	
NQO1*2 (g.559C>T, p.P187S, rs1800566)		3.09
*1/*1 (C/C)	210; 82.3% (77.2–86.6; 0.05)	
*1/*2 (C/T)	40; 15.7% (11.7–20.7; 0.04)	
*2/*2 (T/T)	5; 2% (0.7–4.6; 0.02)	
*1 (C)	460; 90.2% (87.3–92.5; 0.03)	
*2 (T)	50; 9.8% (7.5–12.7; 0.03)	

^a^ Only those SNPs detected in the analyzed samples from Caribbean Hispanic in Puerto Rico are reported in this table. SNPs in the assay panels [16–21] with non-calls or call rates<95% were excluded.

^b^ Only 84 individuals were tested for this variant, including those who tested positive for CYP2C9*8 in a previous assay (c.-1188T>C and CYP2C9*8 SNPs are deemed to be in strong linkage disequilibrium, LD).

^c^ Fisher Exact test.

Notably, three patients were carriers of the CYP2C9*8 allele (all heterozygous) and another five (5) were carriers of the CYP2C9*5 allele (c.1080C>G transversion in exon 7, p.N360E). The *5 allele has been mainly reported in individuals of African descent and has been early associated with a substrate-dependent decrease in *in vitro* intrinsic clearances for CYP2C9 pathway that ranges from 8 to 18% of wild-type [39–40]. Additionally, the *8 allele has been identified in African-American patients [39, 41–42] and also accounts for a decrease in CYP2C9 enzyme activity of 30% for S-warfarin [43]. Moreover, the CYP2C9*6 allele (g.10601delA; p.K273X) was identified in two subjects of this study cohort (both heterozygous for this variant, but one of them showing a *3/*6 double-carrier status, patient# WPR255); also the rare variants CYP2C9*4 (g.42615T>C, p.I359T, *rs56165452*) and CYP2C9*11 (g.42542C>T, p.R335W, *rs28371685*) were detected in four and one additional participants, respectively (all heterozygous). Another rare variant found in these patients was the c.-1188 T>C SNP in the promoter region of the *CYP2C9* gene (MAF = 0.357; frequencies of genotypes C/T: 0.405 ±0.31–0.51, Margin of Error = 0.10 and C/C: 0.155 ±0.09–0.25, Margin of Error = 0.08; HWE test χ^2^ = 1.18), but not the c.-1766 T>C polymorphism (rs9332094). Notably, both of them are often in strong linkage disequilibrium with the *8 allele [44].

The frequencies of the CYP4F2*3 (g.18000G>A, p.V433M, *rs2108622)* and NQO1*2 (g.559C>T, p.P187S, *rs1800566)* “resistant” polymorphisms in the study population were 0.11 (95%CI: 0.09–0.14) and 0.10 (95%CI: 0.07–0.13), respectively. There are 18% heterozygous (G/A) for the CYP4F2*3 allele (95%CI: 0.14–0.23) and 2.4% homozygous (A/A) for this variant allele (95%CI: 0.01–0.05); whereas, about 15.7% of patients carried the C/T heterozygous genotype for the NQO1*2 SNP (95%CI: 0.12–0.21) and 2% were double carriers (T/T) of this variant (95%CI: 0.007–0.05). Moreover, one patient (# WPR274) was identified as heterozygous for the “rare” missense variant c.134T>C transition in the *VKORC1* gene (*rs104894540*), which is an autosomal dominant warfarin resistance genotype resulting in a valine-to-alanine substitution at codon 45 (p.V45A). Another eight (8) participants are carriers of the H7 “resistant” haplotype, including the homozygous H7/H7 patient # WPR265.

### Validation Cohort

Subjects enrolled in the external “independent” validation cohort (n = 55) are younger (mean age: 60.7 years; range: 23–90 years) and included more female (approx. 64%) than those in the derivation sample. Sixty percent (60%) had AFib as their primary indication for therapy with warfarin. The other clinical and demographic variables are fairly well balanced between the two cohorts (Table 1). About thirty-six percent (36.4%) of subjects in this cohort are wild-types, 11% are single carriers of polymorphisms on either *CYP2C9* (9%) or *VKORC1* (2%); 5.5% are double carriers for *CYP2C9* variants (including one *2/*3) and 34.5% are *VKORC1*-1639 AA; whereas, 9% are triple carriers for variants on both *CYP2C9* and *VKORC1* (i.e., CYP2C9*1/*2 + *VKORC1* AA) and 3.6% are quadruple carriers (i.e., CYP2C9*2/*2 + *VKORC1* AA).

### Model Development

**Fig 2:**
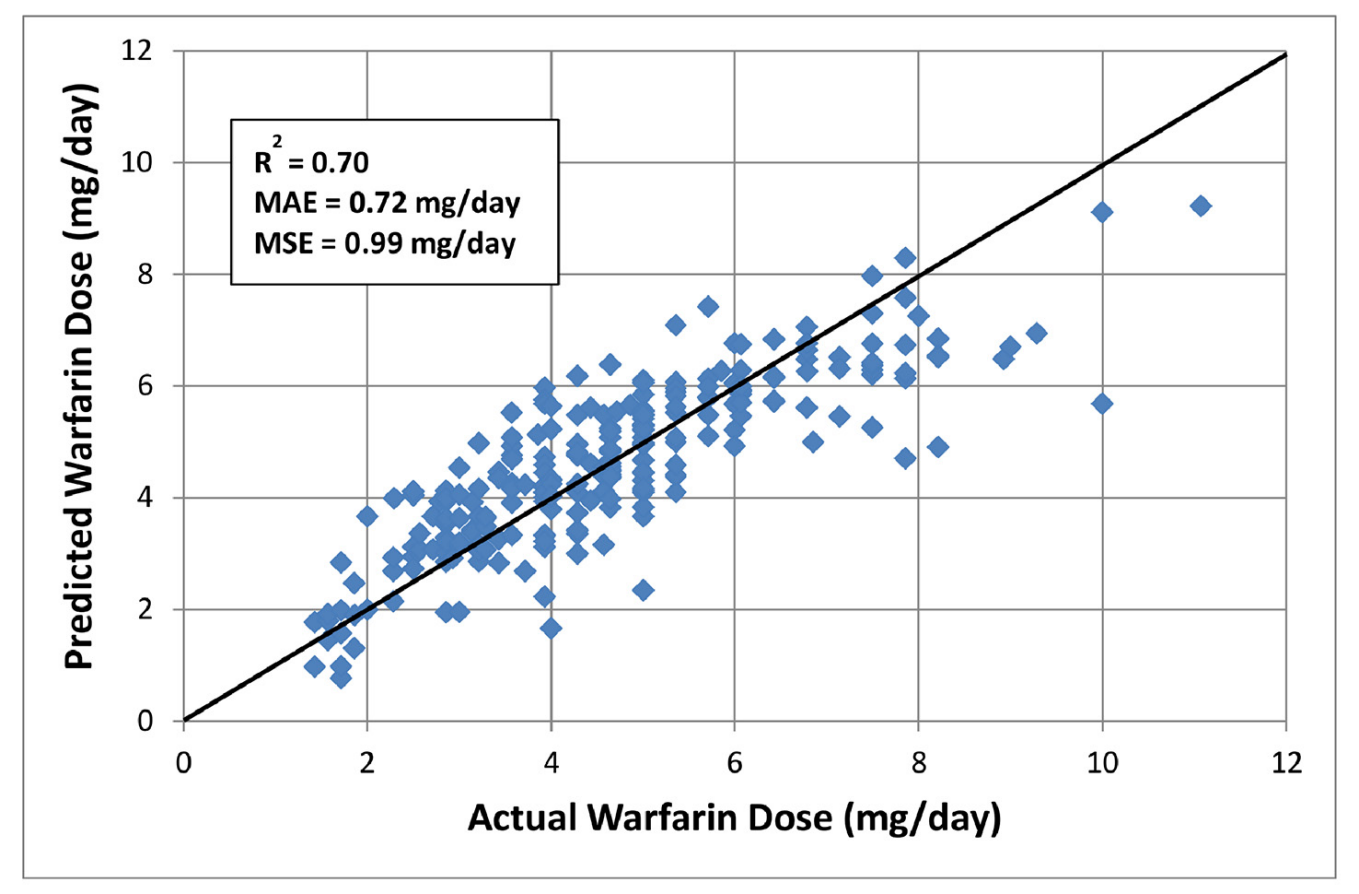
Admixture-adjusted pharmacogenetic warfarin dose refinement algorithm in Caribbean Hispanic patients, developed by using a multiple regression analysis in a derivation cohort of 255 individuals. The solid “identity” line illustrates perfect prediction. MAE and MSE stand for the mean absolute error and the mean standard error of estimate, respectively.

The respective regression coefficients, p-values, the percent of effect on dose requirements and the contribution to explain observed variability that is accounted for by the model, as measured by the partial R^2^ statistics, are presented in Table 3 for all the variables that were entered into the regression model. The performance of this pharmacogenetic model is depicted in Fig 2.

**Table 3:** Summary of attributes of the pharmacogenetic equation for warfarin dosing refinement in Caribbean Hispanics. Derivation cohort (n = 255 patients from the VACHS).

Model Summary					
Variables^a^	Partial Regression Coefficient	Std. Error	Adjusted-R^2^ after entry	Effect on warfarin dose ^b^	*p*-value
Constant	3.246	0.656			
Age	-0.0181	0.0067	0.049	-5%	0.008
D1	0.425	0.0488	0.463	13%	<0.0001
tINR	0.516	0.253	0.471	15%	0.043
Dose-adjusted INR^c^	-1.419	0.1437	0.591	-11%	<0.0001
CYP2C9*2	-0.245	0.1654	0.613	-7%	0.014
CYP2C9*3	-0.544	0.2224	0.625	-16%	0.015
CYP2C9*8	-1.00	0.6014	0.638	-31%; -18%^d^	0.098
*VKAA*	-0.896	0.2169	0.641	-12%	<0.0001
*VKGA*	-0.379	0.1391	0.659	-14%; -13%^e^	0.007
CYP4F2*3	0.560	0.2274	0.673	17%	0.014
NQO1*2	0.346	0.2246	0.689	10%	0.125
Admixture	-0.093	0.0577	0.694	-7%	0.108
Amiodarone	-0.795	0.4173	0.699	-24%	0.06

^a^ Variables are listed in the order they were incorporated into model using stepwise regression analysis.

^b^ Effect (%) on the estimates of the effective dose is calculated per number of variant alleles (*CYP2C9*, *CYP4F2*, *NQO1* and *VKORC1*), per decades (Age) and per 0.25-unit increase in the dose-adjusted INR response at 3^rd^ day.

^c^ INR over dose at 3^rd^ day. Age in years-old; *VKORC1-*1639G>A status: *VKAA*, carriers of A/A genotype; *VKGA*, carriers of G/A genotype; *CYP2C9*, carriers of *2, *3 or *8 polymorphisms on the *CYP2C9* locus; Amiodarone, patients who are taking amiodarone concomitantly. D1 is the initial dose of warfarin (mg/day) on Day 1 of therapy. tINR stands for the middle value of the target INR range, as per indication to warfarin (e.g., 2–3 for Afib is entered as 2.5).

^d^ Effect on warfarin dose of all the three *CYP2C9* polymorphisms combined (i.e., *2, *3 and *8).

^e^ Effect on warfarin dose of both *VKAA* and *VKGA* status combined.

Combined *VKORC1*-1639AA and GA genotypes explained approximately 2.1% of the dose variability in this population. Moreover, this covariate was associated with a 13% reduction in the therapeutic warfarin dose per number of A alleles. The number of loss-of-function *CYP2C9* polymorphisms combined explained another 5% of the observed inter-individual variability in dose requirements, and it was associated with an average of 18% decrement in the warfarin dose per variant allele. Notably, CYP4F2*3 and NQO1*2 variants were independently associated with a 17% and 10% increase of the dose per variant allele, respectively; whereas, the admixed status of the admixture index decreases the dose by 7%. None of the tested *EPHX1* variants entered into the final model. Dose-adjusted INR at day 3 was one of the non-genetic clinical variables to enter the model, and each 0.25-unit increase in this interaction variable was associated with an 11% decrease in the therapeutic warfarin dose. This variable explained approximately 12% (95% CI: 6.3–25.6) of the observed dose variability. Other factors that entered the regression model were age, dose at day 1, target INR range and co-therapy with amiodarone. Overall, this admixture-adjusted pharmacogenetic model in Caribbean Hispanics contained thirteen significant ‘explanatory’ variables. These independent predictors explained more than two-thirds of the observed variance in the therapeutic warfarin dose (R^2^ = 70%; MAE = 0.72mg/day; MSE = 0.99; p< 0.001), which is better than that of a clinical non-genetic algorithm in the same derivation cohort (R^2^ = 60%; MAE = 0.99mg/day; MSE = 1.26). Interaction terms between the genotypes and key co-medications (e.g., amiodarone, statins, azoles and sulfamethoxazole) were not statistically significant.

A polynomial regression model was discarded as the distribution did not suggest a curvilinear relationship between warfarin doses and the model variables. A multiple, least-squares, linear regression model proved to be the best option for the available data, according to the lowest MAE criterion. The stepwise regression analysis yielded the following PGt algorithm to predict warfarin doses in Hispanic Puerto Ricans (as seen in Fig 2):

$$Warfarin\;Dose\;\left(\frac{mg}{day}\right)=\left[3.2463-(0.0181∙Age)+(0.4246∙D1)+(0.5157∙tINR)-(1.419∙\frac{INR}{D3})-(0.2450∙{CYP2C9}^{*}2)-(0.5442∙{CYP2C9}^{*}3)-(1.00∙{CYP2C9}^{*}8)-(0.3793∙VKORC1GA)-(0.8958∙VKORC1AA)+(0.5602∙{CYP4F2}^{*}3)+(0.3457∙{NQO1}^{*}2)-(0.7948∙Amiodarone)-(0.0932∙Admixture)\right]$$ (1)

Where INR/D3 is the corresponding ratio of the dose-adjusted INR measures on day 3 of initiation of warfarin therapy; D1 is the initial dose of warfarin (mg/day) on Day 1 of therapy; tINR stands for the middle value of the target INR range, as per indication to warfarin (e.g., 2–3 for Afib is entered as 2.5); *VKORC1* AA status is a carrier code, where 1 = AA and 0 = otherwise; *VKORC1* GA status is a code, where 1 = GA and 0 = otherwise; all *CYP2C9* status, as well as the CYP4F2*3 and the NQO1*2 status, are codes, where 0 = wild-type, 1 = one mutated allele and 2 = two mutated alleles; amiodarone user code = 1 if the patient is taking that drug and 0 = otherwise; and age is in years. Finally, the admixture index code is as follows: 1 = European ancestry, 2 = Native American ancestry, 3 = African ancestry, 4 = Admixed individual.

To select candidate variables for the multiple linear-regression model of warfarin dosing prediction in Caribbean Hispanics, we initially examined the independent effects of demographics (i.e., age, gender, height and weight or body mass index), clinical (i.e., treatment indication, comorbidities, use of co-medications [amiodarone, antifungal azoles, statins, Bactrim], target INR range, initial doses (days 1 thru 3 of therapy), dose-adjusted INR at day 3, diet and smoking status) and genetic variables (i.e., *VKORC1* genotypes, number of *CYP2C9* variants, CYP4F2*3 18000G>A (V433M, *rs2108622)* and NQO1*2 c.*559C>T (P187S*, *rs1800566)* carrier status) individually.

The admixture-driven pharmacogenomic algorithm developed in Caribbean Hispanics predicted effective warfarin dose with R^2^ values of 63%, 50% and 20% for low, intermediate and high-dose groups, respectively (Table 4). These R^2^ values surpassed predictability of the clinical non-genetic algorithm for the same dosing groups in the derivation cohort of Caribbean Hispanic patients (i.e., 50%, 45% and 2%, respectively). As expected, our model’s predictability also outperformed both the Lenzini’s and the IWPC’s algorithms in this study cohort (i.e., Lenzini’s model: 24%, 20%, and 7% and IWPC’s model: 23%, 13% and 2% of variability explained for the same dosing groups, respectively). Furthermore, in more than half of patients the MAE value was <1 mg/day (i.e., falling within 20% of the actual dose, which is a well-accepted criterion for accuracy in dose estimation).

**Table 4:** Warfarin daily dose predictability of the admixture-adjusted pharmacogenetic refinement model developed in Caribbean Hispanics (CH), the Lenzini *et al*. pharmacogenetic refinement model [36], the IWPC pharmacogenetic initiation algorithm [37] and the clinical algorithm^a^ as compared with the actual doses of warfarin for the therapeutic effect in patients requiring low (≤3 mg/day), intermediate (>3 and <7 mg/day) or high (≥7 mg/day) exposure. Study cohort of 255 warfarin patients at the VACHS anticoagulation clinic.

Prediction Model	IWPC Algorithm	Lenzini Algorithm	Clinical Algorithm^a^	CH Algorithm
**Low Doses (≤3 mg daily)**				
MAE (mg/day) 95% CI	1.27 (1.02–1.52)	0.63 (0.49–0.77)	1.22 (1.05–1.39)	0.51 (0.39–0.63)
% bias (precision)	53%	26%	50%	21%
R^2^ (%)	23%	24%	50%	63%
**Intermediate Doses (>3 and <7 mg daily)**				
MAE (mg/day) 95% CI	0.97 (0.85–1.09)	1.2 (1.08–1.32)	0.83 (0.74–0.92)	0.61 (0.53–0.69)
% bias (precision)	21%	26%	18%	13%
R^2^ (%)	13%	20%	45%	50%
**High Doses (≥7 mg daily)**				
MAE (mg/day) 95% CI	2.48 (2.03–2.93)	3.20 (2.73–3.67)	1.53 (1.03–2.02)	1.53 (1.17–1.89)
% bias (precision)	31%	40%	19%	19%
R^2^ (%)	2%	7%	2%	20%

MAE stands for mean absolute error. The 95% confidence intervals (CIs) on the estimates of MAE were calculated.

^a^ This algorithm is the same model developed in Caribbean Hispanics but excluding genotypes and admixture. R^2^ is the coefficient of determination.

For patients at the highest risk who required either ≤3mg (~19.2% of the study cohorts) or ≥7 mg/day of warfarin per day (~11% of the study cohorts), 54.5% of the dose predictions using our developed model resulted within 20% of the actual dose (‘ideal dose’) as compared with 29% when using the clinical non-genetic algorithm (p<0.001), 34% with the Lenzini’s model (p<0.001) and 24.7% with the IWPC’s algorithm (p<0.001). Similarly, for patients requiring between 3 and 7 mg/day (intermediate-dose group: 69.8% of the total cohort), our admixture-adjusted pharmacogenetic algorithm predicted doses in the ideal range for significantly more patients than the clinical algorithm or the two earlier published pharmacogenetic models (i.e., 78 vs 61, 39 and 62%, respectively; p<0.001 for each comparisons, Table 5).

**Table 5:** Predictions of ideal doses by using the admixture-adjusted pharmacogenetic refinement model developed in Caribbean Hispanics (CH), the Lenzini *et al*. pharmacogenetic refinement model [36], the IWPC pharmacogenetic initiation algorithm [37] and the clinical algorithm^b^ in patients requiring low (≤3 mg/day), intermediate (>3 and <7 mg/day) or high (≥7 mg/day) exposure. Ideal dose is defined as within 20% of the actual warfarin dose at stabilization.

Model	Ideal Dose (%)	Underestimated (%)	Overestimated (%)	p-value^a^
**Low Doses (≤3 mg daily)**				
IWPC algorithm	24.5	4.08	71.43	0.008
Lenzini model	44.9	8.20	46.9	0.48
Clinical algorithm^b^	10.20	0.00	89.80	0.0001
CH Algorithm	53.06	2.04	44.9	-
**Intermediate Doses (>3 and <7 mg daily)**				
IWPC algorithm	62.4	18.54	19.1	0.0021
Lenzini model	39.3	53.9	6.74	0.0001
Clinical algorithm	61.8	2.8	35.39	0.0005
CH Algorithm	77.0	8.43	14.61	-
**High Doses (≥7 mg daily)**				
IWPC algorithm	25.00	75.00	0.00	0.0159
Lenzini model	14.29	85.71	0.00	0.006
Clinical Algorithm	60.7	39.3	0.00	0.68
CH Algorithm	57.14	42.86	0.00	-

^a^ *p*-values for the comparison of proportions of “ideal dose” estimates using the admixture-adjusted pharmacogenetic algorithm versus the clinically-based, the Lenzini’s and the IWPC-derived algorithms. McNemar’s test of paired proportions was used, with significant differences set at *p<0*.*05*.

^b^ This algorithm is the same model developed in Caribbean Hispanics but excluding genotypes and admixture.

In general, the admixture-based pharmacogenomic algorithm tailored specifically for the Caribbean Hispanic population yielded consistently better dose predictions as compared to the Lenzini’s and IWPC’s algorithms. In particular, we observed good predictions of ideal doses in ~70% of the entire study cohorts when using our model, with significantly fewer under- and overestimations of the ideal doses (11% and 19%, respectively). In contrast, the Lenzini’s model barely predicted ideal doses in less than a half of subjects that came up with good predictions after using our model (i.e., ~38%, 96/255); whereas, the IWPC’s model only predicted correctly the ideal dose in approx. 51% of participants.

**Fig 3:**
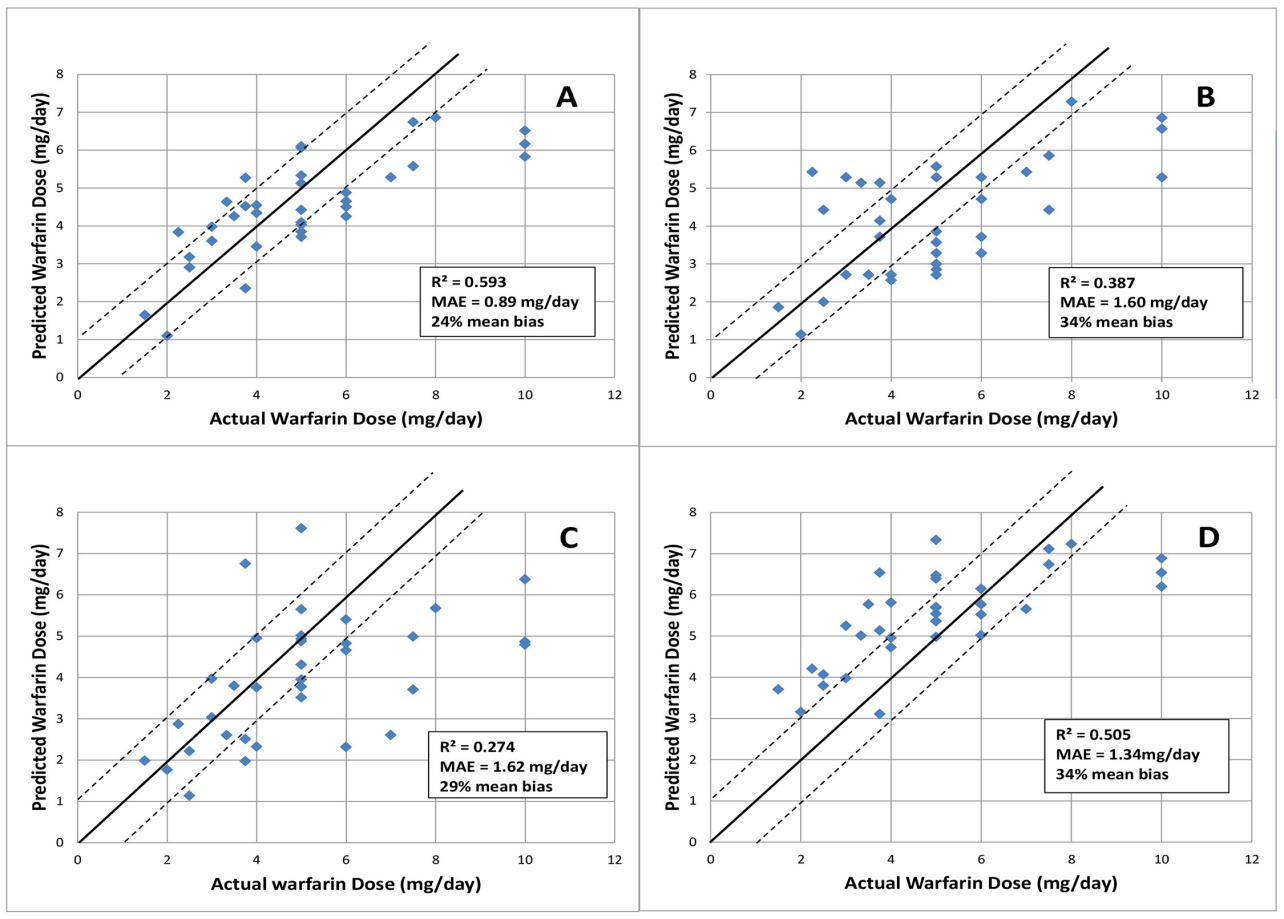
Validation of the admixture-adjusted pharmacogenetic algorithm for dose refinement in Caribbean Hispanics (upper plot A) as compared to two publicly available algorithms (i.e., IWPC-derived [37] and Lenzini *et al* [36] models, plots B and C, respectively) as well as a clinical non-genetic algorithm (plot D) in an independent sample of 55 Puerto Ricans from the Brownstone Clinic in Hartford, CT. Each filled diamond represents the observed versus predicted dose of each patient. The upper solid line is (predicted + 1 mg/day) of the actual dose, the middle solid line (i.e. 45% degree line) illustrates perfect prediction in this validation cohort, and the lower solid line is (predicted −1 mg/day) of the actual dose. 1 mg/day change in warfarin dose is sufficient to change the INR by 0.5, a clinically meaningful difference.

A correlation plot of the predicted warfarin daily dose based on the admixture-driven pharmacogenetic algorithm developed for Caribbean Hispanics as compared with the actual daily dose in the validation cohort is shown in Fig 3A. As can be observed, our pharmacogenetic model predicted 59% of the variance in warfarin dose when externally validated in 55 genotyped individuals from the independent Hartford’s patient cohort (MAE = 0.89 mg/day, 24% mean bias). On the contrary, the two others reference PGt algorithms showed poorer dose predictability in this independent cohort. Fig 3B and 3C depicts the corresponding statistics for these algorithms (i.e., IWPC’s model: R^2^ = 39%, MAE = 1.60 mg/day, 34% mean bias; Lenzini’s model: R^2^ = 27%, MAE = 1.62 mg/day, 29% mean bias). On the other hand, the clinical algorithm that excluded genotypes and admixture only accounted for 50% of dose variability in this independent cohort (MAE = 1.34 mg/day, 34% mean bias, Fig 3D).

**Fig 4:**
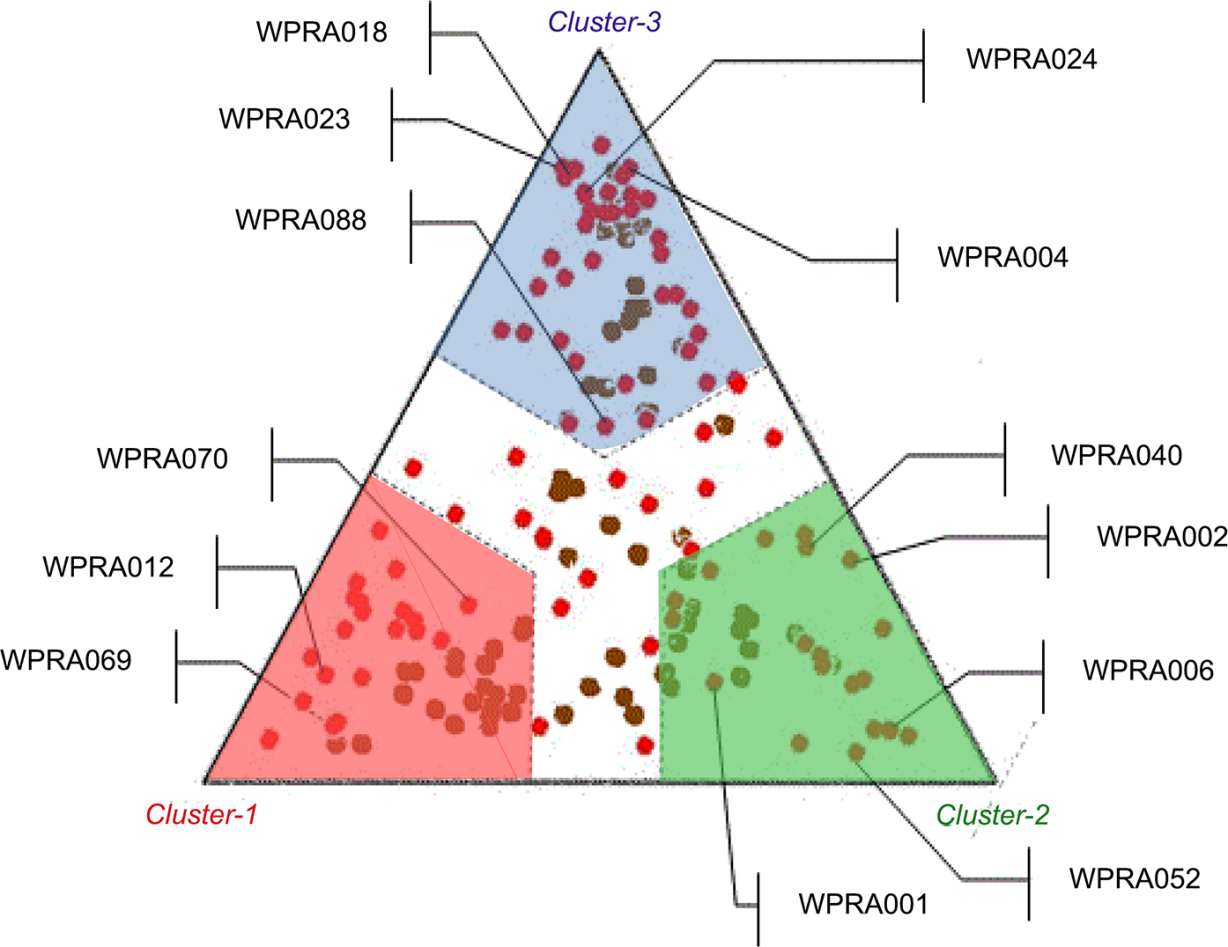
Association between the degree of Amerindian ancestry and low dose requirements. ID codes depicted for those requiring <3mg/day.

Fig 4 depicts a typical STRUCTURE-derived triangular landscape with ancestry-specific vertices (i.e., European, African and Native-American in a counterclockwise sense) on which we plotted the results of the Bayesian’s clustering analysis used to measure individual admixture proportions of participants (i.e., the ones used for the algorithm derivation). Notably, a portion of these individuals (red dots) are scattered throughout the triangle area (i.e., not clearly positioned toward any of the vertices but displayed in the middle of the triangle), showing hence a marked admixture. We found that the Native-American sector in the right-most vertex of the SRUCTURE triangle (in green) showed a higher proportion of patients with low-dose requirements (<3 mg/day) than the rest of the clusters (i.e., 33% vs. 19%, p<0.01).

## Discussion

The sampled cohort is representative of the target population. The observed frequency distribution of multiple ethno-specific polymorphic variants in these warfarin-related pharmacogenes confirmed a richer genetic diversity in this highly admixed population of Caribbean Hispanics as compared to other parental groups like Caucasians. Noteworthy, some of these variants have been previously described exclusively in either people of African ancestry (e.g., *5,*6,*8, *11) or Caucasians (e.g., *2,*3), but not often coexisting in both of them. Besides, the combination of CYP4F2*3 and NQO1*2 “resistant” variants, identified in as many as 20.4% and 17.7% of patients in the study cohort, had only been previously described to be associated with higher warfarin dose requirements in another US Hispanic population [12]. *CYP4F2* gene encodes cytochrome P450, family 4, subfamily F polypeptide, a member of the cytochrome P450 superfamily of enzymes that is involved in vitamin K1 metabolism. The NAD(P)H dehydrogenase, quinone 1 (*NQO1*) gene encodes for a FAD-binding cytoplasmic 2-electron reductase enzyme that catalyzes the reduction of vitamin K to its active hydroquinone form, and is therefore involved in the vitamin K-dependent prothrombin synthesis.

Most of existing pharmacogenetic-guided algorithms include only the CYP2C9*2, *3 and *VKORC1*-1639G>A variants commonly found among Caucasians [45–52]. Uncommon mutations in Europeans (e.g., CYP2C9*8, NQO1*2) are often overlooked and, consequently, the utility of existing algorithms is limited in patients with mixed ancestry like Caribbean Hispanics. A recent clinical trial (COAG, NCT00839657) raised some concerns about clinical utility of genotyping in warfarin dose predictions [53]; however, we firmly believe that failure to account for variants relevant in underrepresented ethnicities (e.g., African Americans or admixed Hispanics) will lead to significant dosing error in these populations.

Similar to our previous pharmacogenetic-guided algorithm in Puerto Ricans [38], carrier status for *VKORC1*-1639G>A and loss-of-function *CYP2C9* polymorphisms were significant predictors of therapeutic dose in the herein developed admixture-adjusted PGt algorithm of dose-refinement within Caribbean Hispanics. However, the *CYP2C9* carrier status (i.e., combinatorial CYP2C9*2 plus *3 alleles, along with the *8 variant), was this time a better predictor of therapeutic doses in our derivation cohort.

Despite of being the first variable to show significance in the previous algorithm, *VKORC1* GA and AA were now the 8^th^ and 9^th^ variables to enter a stepwise regression model that predicted the refined dose in the new algorithm (Table 3). Interestingly, *VKORC1* genotypes combined contributed only ~2.1% of the total R^2^, which suggests that the INR/dose3 value captured most of the information on warfarin sensitivity in the derivation cohort. A similar observation was earlier noted by Lenzini *et al*. [36] in another dose-refinement model developed in a cohort of mostly Caucasian patients. Moreover, previous reports also found that *VKORC1* correlates with INR response over the first week of treatment; whereas, a strong association with dose vanished during this initial week of therapy [36]. It has been hypothesized by others that *CYP2C9* variants could overcome the predictive effect of *VKORC1* in PGt algorithms for dose-refinements because of their direct impact on S-warfarin metabolism, a process that seems to be more clinically relevant after the first few days. Since INR measure reflects warfarin sensitivity but not warfarin clearance, this value will mask the *VKOC1* effect over time. Nonetheless, the addition of *VKORC1* in the final model is expected to partially reduce the INR effect on dose variability, thus minimizing the impact of errors in initial INR measurements. Lenzini *et al*. have previously acknowledged this expectancy; particularly in patients being treated with unfractionated or low-molecular-weight heparin that sometimes inflate initial INRs [36].

Although the admixture index only explained less than 1% of observed dose variability in this cohort, it represents approximately a 7% of dose decrement for admixed individuals. Accordingly, we strongly believe this variable is a clinically relevant predictor for our model in Caribbean Hispanics given the well-documented stratification of this population in the Island of Puerto Rico and the Caribbean basin at large. An earlier paper by Via *et al*. [54] provides evidence in support of the need for adjustments by admixture degree in pharmacogenetics studies within Caribbean Hispanics in order to control for significant population stratification given its marked ethno-geographic heterogeneity and distinctive clines of admixture proportions across the Island (i.e., people with the higher African ancestry mainly located in the east side of the Island; whereas, individuals who have the greatest European contribution mostly living in the west region).

To the best of our knowledge, no previous trials have included a measure of admixture in warfarin dose predictions, even though a few of them have certainly added ethnicity or race as a proxy [12]. Admixture can be a confounder that may result in false associations due to differences in genetic backgrounds across individuals in the derivation cohort [11,13, 55–56]. Findings depicted in Fig 3 suggest caution on use of pharmacogenetic models in Hispanics without accounting for admixture or ethnic-specific SNPs. Fig 4 shows result of the Bayesian’s clustering analysis of PG array data in order to infer the unique genetic ancestral roots among members of the admixed population under study [27]. Based on this result, we concluded that there is an association between the degree of individual Amerindian ancestry and low warfarin dose requirements. This is not surprising given the fact that people of Asian or Amerindian ancestry are more likely to need lower doses because of their higher *VKORC1* haplotype A frequency. Admixed populations may differ from other populations in frequency, distribution and combination of allelic variants in their genomes [57–59]. There are also important differences in linkage disequilibrium (LD) by ancestry that might have significant consequences for the association between genotypes and warfarin dosing across different populations.

Other variables that were not important predictors of dose revisions in our study cohort were body size, gender, vitamin K intake, statin use, azoles use, smoking status, liver function, diabetes, and indications. Type 2 Diabetes shows high prevalence among subjects in the study cohort and has been earlier reported as a marker for lower warfarin dose requirements [60]. A possible explanation is that these variables were all taken into proper consideration when initial doses were estimated at the anticoagulation clinic and their predictive ability appears to wane over time.

In practice, current turnaround time for genotyping results and ancestral proportions estimation could take ~2–3 days. Such an inherent delay limits our ability to initiate dosing based on pharmacogenetic information. Furthermore, various experts consider genotypes irrelevant once INR measurements become available after some days of therapy ([36]. This apparent lack of explicit cost-benefit for patients is compounded by the poor predictability of prior PGt-guided initiation algorithms in admixed populations like Hispanics. Consequently, it is not surprising the lack of fully endorsement by professional organizations in the field [61–63].

However, there is a well-documented higher incidence of warfarin-related bleeding events during and after the second week of warfarin therapy [45, 64–68]. Accordingly, PGt-guided models that refine warfarin dosing are expected to remain relevant in clinical settings after the first 3–5 doses even though these initial doses were not tailored to genotypes. A dose-refinement PGt algorithm can thus allow us to adjust therapeutic warfarin dose once relevant INR measures are also available. Previous developments of PGt refinement algorithms, including the work by the International Warfarin Dose-Refinement Collaboration (Warfarin DR) group [36], have been successful in determining utility of genotypes in patients from different ethnicities, even after INR levels become available on day 4–5^th^ of therapy, but none in Caribbean Hispanics.

Herein we present results for a newly developed and validated admixture-driven PGt algorithm for warfarin dose-refinement in Caribbean Hispanics, which showed very good performance, accuracy and robustness in predicting therapeutic doses in the target population. Our findings provide further support in favor of the proposed pharmacogenetic approach to predict optimal individual’s warfarin doses, even after 4 days of treatment, as compared to a standard clinical method.

On the other hand, our model outperformed two previous pharmacogenetic-guided algorithms (i.e., a dose-refinement algorithm by Lenzini *et al* [36] and the PGt algorithm for dose initiation developed by the IWPC that evaluate dosing variability at earlier time points [37]), with higher R^2^ (i.e., 0.70 versus 0.34 and 0.27, respectively), less scatter and lower MAE (MAE = 0.72 mg/day versus 1.33 and 1.2 mg/days, respectively), when using dataset from our derivation cohort of 255 patients. Accordingly, these existing pharmacogenetic-guided algorithms derived from other populations (mostly Caucasians) were poor predictors of the actual therapeutic warfarin dose in Caribbean Hispanics, explaining no more than 30% of the total dose variation in Caribbean Hispanics as compared to ≥ 60% in Caucasians [36–37].

Likewise, the developed algorithm also performed better for the Caribbean Hispanic population than a clinical algorithm that excludes genotypes and admixture (R^2^ = 0.6 and MAE = 0.99 mg/day). After adjusting for INR at 3^rd^ day and admixture, clinical polymorphisms on major warfarin-related pharmacogenes (*CYP2C9* and *VKORC1*) remained significant predictors of effective dose (p<0.05). Therefore, this admixture-driven PGt algorithm estimates effective warfarin doses after 4 days of therapy more accurately than the one that only used clinical factors.

Analysis in Table 5 shows no significant difference between our admixture-adjusted PGt model to predict optimal doses in the high-dose subset of patients (≥7 mg/day) and the clinical non-genetic algorithm (p = 0.683), but the admixture-adjusted PGt algorithm was significantly more accurate in the subsets with the intermediate (p = 0.0005) and low-dose requirements (p = 0.0001). We consider that our PGt algorithm is particularly more useful in patients who are carriers of the “high-risk” unusual genotypes. As a matter of fact, 54% of dose predictions in patients at the highest risk for poor outcomes (i.e. ≤3mg or ≥7 mg/day) resulted in ideal doses with our developed model, as compared with only 29% with the clinical algorithm. Previous reports indicate that algorithms guided by genetic information are able to correct the genetic propensity to high INRs in individuals with variant SNPs [68].

Although one study limitation is the single-center nature of the study design, the fact that all participants were managed by the same anticoagulation services (i.e., clinicians, procedures, formulary, dosing strategies, CPRS data entry, etc.) minimizes differential managements that could represent a potential bias to account for variability in dose requirements. Important to remark that the San Juan VACHS-affiliated anticoagulation clinic is the main and largest anticoagulation center in the Caribbean area, with patients from all ethno-geographic regions of the Island of Puerto Rico that ensure representativeness. Nonetheless, potential biases remain in any non-randomized study. Accordingly, we propose this algorithm to be investigated in a larger randomized, prospective clinical study in Caribbean Hispanics. Other limitations of the study include gender and elderly, as well as issues related to the observational and retrospective nature of the study design. All these limitations are also valid for the clinical non-genetic algorithm.

In general, our findings in Caribbean Hispanics are consistent with those reported in previous studies of warfarin PGt in other populations to predict dose-refinements, including those by Lenzini *et al*. and Horne *et al*. [36, 68]. The addition of genotypes and admixture improves the R^2^ by ~10%. The absolute difference in predicted vs. observed doses in the overall patient cohort was 0.72 mg/day, which corresponds to a 14.4% of mean absolute percentage of difference (precision). This prediction error of only 14% represents a difference of <1 mg/day relative to the traditional starting dose of 5 mg/day, which clinicians define as not clinically relevant. By systematically using this novel admixture-adjusted PGt algorithm, we anticipate that clinicians could now estimate a more accurate “tailored-to-genotype” therapeutic warfarin dose in Caribbean Hispanics after four days of therapy that translate into a safer and more effective therapy, with those at the highest-risk likely gaining the most benefit from this approach.

A multi-center, randomized clinical trial (ClinicalTrials.gov Identifier: NCT02345356), aimed at comparing our admixture-adjusted PGt algorithm for warfarin dose predictions in Caribbean Hispanics versus current standard of care, will be conducted soon in order to evidence its clinical utility.

## Supporting Information

S1 Text Copy of the IRB-approved clinical protocol for the study related to this work. (PDF)

S2 Text Copy of the Informed Consent Form, English version. (PDF)

S3 Text Copy of the HIPAA Authorization Form. (PDF)

S4 Text TREND Statement Checklist. (PDF)
