# Supplementary material for: A Novel Admixture-Based Pharmacogenetic Approach to Refine Warfarin Dosing in Caribbean Hispanics
Source: PLoS One. 2016 Jan 8;11(1):e0145480. doi: 10.1371/journal.pone.0145480 (PMC4706412; doi:10.1371/journal.pone.0145480)
Supplement: S1 Text — (PDF) [file pone.0145480.s001.pdf]

RESEARCH PROTOCOL TO BE REVISED BY IRB

PHARMACOGENETIC-DRIVEN WARFARIN DOSING ALGORITHM IN PUERTO RICANS

PROTOCOL VERSION #6
INCLUDES AMENDMENT #4
SUBMITTED: NOV 30, 2011

Abstract:

Warfarin is a widely used anticoagulant with a narrow therapeutic window. Despite careful monitoring Prothrombin Time (PT) by International Normalized Ratio (INR), bleeding and thrombotic complications are common, resulting in substantial morbidity, mortality and cost. In patients starting anticoagulant therapy, determination of the appropriate therapeutic maintenance dose of warfarin may require several weeks or months of trial and error, placing the patient at risk and requiring excessive clinical resources. Genetic factors are now recognized to significantly influence warfarin dose and metabolism, adding to traditional factors currently weighted by clinicians, including age, gender, height, weight, diet (vitamin K intake), and concomitant medications. Common variants of the cytochrome P450 2C9 gene (*CYP2C9**2 and *3 mutations) decrease warfarin metabolism while A>G mutations in the promoter region (-1636) of the *VKORC1* gene, which encodes for the Vitamin K epoxide receptor complex-1, the binding protein for warfarin, alter warfarin sensitivity and dose. Together, these two genetic markers account for approximately 40-50% of warfarin metabolism and action in various populations. These markers in addition to a limited number of environmental determinants explain 50-60% of the variability in warfarin sensitivity in non-Hispanic populations. Evidence suggests that alterations in the dietary intake of vitamin K can affect anticoagulation response to warfarin. It is possible that a low and erratic intake of dietary vitamin K is at least partly responsible for the variable response to warfarin in patients with unstable control of anticoagulation. According to an early report by Sconce *et al* (47-48), daily supplementation with oral vitamin K in unstable patients could lead to a more stable anticoagulation response to warfarin. Indeed, these authors found that the mean daily intake of vitamin K in unstable patients was considerably lower than that for stable patients during the study period (29±17μg vs. 76±40μg).

This study proposes to determine combinatorial *CYP2C9*, *CYP4F2, GGCX, NQO1, EPHX1* and *VKORC1* genotypes and to assess genotype-phenotype associations in 300 eligible patients, currently receiving warfarin in the Veterans Administration Caribbean Healthcare System (VACHS) located at their main medical center in San Juan, Puerto Rico. We will test the effect of genotype on warfarin-related clinical outcomes and the warfarin dose by using available DNA-typing assays (e.g., xMAP Luminex technology, TaqMan SNP assays, DNA sequencing, PG-arrays and/or DMET chip assays). We will also ascertain how they compare to others reference populations considering the heterogeneous ethno-geographic heritage of Puerto Ricans. Assuming significant dose-dependent effects on anticoagulation-related endpoints, we will combine DNA typing results with time-to-therapeutic responses and bleeding events, retrospectively collected from medical records, in order to demonstrate association with these clinical phenotypes as well as to explain variability in stable warfarin dosage in Puerto Ricans. Accordingly, genotypes along with some relevant demographic and physiological determinants will then be use to predict optimal warfarin dose by using regression models. The expected outcome is the development of a clinical algorithm that help us predict optimal therapeutic warfarin dose in

the target population (Puerto Ricans, particularly VACHS main medical center outpatient population), decreasing the time to achieve the desired steady-state warfarin levels (time-to-onset) for patient's anticoagulant stabilization. VACHS main medical center ambulatory patient population is >90% Hispanic. To our best knowledge, the frequency of the genetic mutations in this population is unknown.

A secondary endpoint of this study was established to evaluate the relationship of dietary intake of vitamin K to the anticoagulation response to warfarin in unstable Puerto Rican patients compared to those with stable control of anticoagulation as well as to determine whether the VKORC1-1639 G>A polymorphism is either a confounder or an effect modifier of such relationship. About 35 eligible patients (INR-unstable patients), currently receiving warfarin at the Veterans Administration Caribbean Healthcare System (VACHS)-affiliated anticoagulation clinic located at their main medical center in San Juan, Puerto Rico, will be recruited for this specific purpose. These 35 patients will be genotyped for VKORC1 polymorphisms and separated into two groups: unstable patients who are non-carriers of VKORC1 genetic variants (wild-type or control reference group A) and unstable patients who are carriers of VKORC1 genetic variants (test group B). This secondary endpoint will contribute to fill a gap in the existing knowledge of the relationship between dietary intakes of vitamin K to warfarin response in Caucasian patients with unstable control of anticoagulation by providing similar data for a Hispanic population (i.e., Puerto Ricans).

To assess vitamin K intake, each patient will be provided with a vitamin K-oriented intake food questionnaire, with a previously developed food scale and weighing factors, and the same set of detailed instructions on how to complete the questionnaire in order to maximize the consistency and accuracy of dietary recording. A number of previously validated photographs of commonly eaten foods will be included with the questionnaire and used to estimate portion sizes. At the end of each week the patients will be seen at the clinic, where dietary recall approaches will be used when incomplete descriptions of food are reported. If a patient is unable to give details about either the weight or portion size then the weight of food eaten will be approximated using average portion sizes as early described by Sconce *et al* (47-48). The expected benefits for VACHS and other institutions serving the Hispanic (Puerto Ricans) population are 1) to improve patient safety (decreased bleeding complications), and 2) to reduce clinical costs (fewer INR tests, reduce length of stay during warfarin therapy initiation, reduced number of hospitalizations related to warfarin therapy complications and adverse events as well as savings of clinical time). This study will foster the necessary development of a useful DNA-guided Personalized Medicine approach for the Caribbean Healthcare System – Veteran Affair Hospital and the MSC-UPR (School of Pharmacy), potentiating their leadership in this clinical evolving area. Finally, this project will provide essential preliminary data for securing external fund by grant application to the NIH/NHLBI, which plans to sponsor a large prospective study of genotype-enhanced warfarin dosing, and to foundations (such as Robert W. Johnson) supporting studies of the alleviation of health disparities in underserved populations.

RESEARCH PLAN

Background

According to The World Health Organization, and the National Center for Health Statistics (NCHS), Puerto Rico ranks 12 worldwide in cardiovascular disease and strokes deaths, particularly in female ages 35-74 (www.who.int/research/en/). In addition, the legislative assembly of the Commonwealth of Puerto Rico enacted the law No. 4, as of March 19th 2005, P. del S. 211, in order to annually celebrate on March, and quote: "the month for the prevention of deep vein thrombosis (DVT) in Puerto Rico" seeking to increase people's awareness of this serious health problem as well as to educate and prevent them from being victims of such a condition. As can be read thereof, 2 million of individuals suffer from DVT every year in USA and the related pulmonary embolism (PE) is considered a major cause of death at hospitals in the island. The document also states that DVT is a critical problem of public health in Puerto Rico.

Warfarin (Coumadin®) anticoagulant therapy is commonly prescribed for prophylaxis of coagulopathies such as Factor V-Leiden and therapy of patients with a wide variety of venous and arterial thromboembolic disorders, including those with atrial fibrillation and other cardiac arrhythmias, surgical cardiac valve replacement, thrombophlebitis with or without PE, and those at risk of stroke (1). Over 20 million warfarin prescriptions are written per year in the United States, making it the 11th most prescribed drug in the United States (2). Approximately two million new prescriptions are written annually, many for anticoagulant-naïve patients (2). This figure will rise as the elders become a substantial percentage of the population and atrial fibrillation becomes a prevalent medical condition in advanced age adults. Warfarin narrow therapeutic window requires careful titration toward patient's maintenance dose. Difference in successful outcomes during warfarin therapy is a multi-factorial issue, including variables other than treatment itself. Many clinical and behavioral factors influence the response to warfarin and need to be considered when prescribing the drug. These include diet, alcohol intake, body mass, concomitant drug treatment, co-existing diseases, and patient compliance with drug regimen and diet restrictions. Genes are also important. The determination of the initial dose is usually empiric and does not routinely take in consideration genetic factors that affect warfarin sensitivity.

The most feared adverse effect associated with warfarin therapy is bleeding. Warfarin-related bleeding is injurious to patients and costly. Major and fatal bleeding events occur at a rate of 7.2 and 1.3/100 patient years, respectively, according to a meta-analysis of 33 studies (3). With a cost of $13,500 per serious bleeding episode often requiring hospitalization (2),

the aggregate annual health care cost in the United States is $1.15 billion (2). In addition, warfarin therapy is associated with 43,000 adverse drug effects and 17,000 strokes annually (2). Current approaches to warfarin induction fail to prevent adverse events. The risk of overdose is increased now that most patients begin warfarin in the outpatient setting, where daily International Normalized Ratio (INR) monitoring is not feasible (except by means of recently introduced patient self-monitoring). The major flaw of existing warfarin dosing algorithms is that most of them rely on trial-and-error approaches and do not consider individual genetic, patient characteristics and clinical factors. The Anticoagulation-Advisor, published in 2001 by Hudson Physician Communications Inc., and endorsed by The Anticoagulation Forum, includes a guide for evidence-based warfarin initiation following the Crowther's protocol (4). This modified version considered a dosage reduction by half for patients who are frail, aged, malnourished, receiving broad-spectrum antibiotics therapy or having liver disease. Even though most previous studies concluded that warfarin requirements are less for the elderly, only the Roberts protocol considered dose adjustments for aged patients (5-9). Patient characteristics and clinical influencing factors on warfarin initiation and maintenance dosages have not uniformly been considered by all trials nor has the individual's unique genetic make-up.

To reduce the risk of overdose and hemorrhage during warfarin induction, experts advocate prescribing the anticipated therapeutic dose to patients who are beginning warfarin therapy. Nevertheless there has been no accurate way to estimate that dose. Recent developments indicate that by means of Pharmacogenetics-based warfarin therapy, clinicians could estimate the therapeutic dose (a priori) by genotyping their patients for single nucleotide polymorphisms (SNPs) that affect warfarin metabolism or sensitivity. Hence, Pharmacogenetics-based therapy might reduce medical expenditure by reducing patient's length of stay (LOS) at the hospital until their therapeutic dose has been determined empirically.

The FDA Office of the Commissioner claimed for collaboration to develop a genetic algorithm to significantly reduce serious adverse effects of warfarin (10). Cytochrome P450 2C9 isoform gene, *CYP2C9**2 and *3 alleles are associated with reduced enzymatic activity, and mutations in the Vitamin K epoxide reductase complex 1 (*VKORC1*) gene cause warfarin sensitivity and multiple coagulation factor deficiency type 2. Patients with either of these genetic variations should receive a warfarin starting dosage of less than 5 mg/day (11). About 55% of the variability in warfarin dose – INR relationship could be explained by the combination of *VKORC1* and *CYP2C9* genotypes, age, height, body weight, interacting drugs, and indication for warfarin therapy in Caucasian patients. Similar observations have been reported in Asian patients (12-15).

The individual's unique genetic make-up is becoming increasingly clear to play a cardinal role in pharmacokinetic (metabolism) and pharmacodynamic of warfarin. *CYP2C9* and *VKORC1* genetic variants are common and have been shown to predict significant variation in warfarin sensitivity in Swedish, English, Japanese, Italian, North American and Hong Kong Chinese populations typically in cohorts of 100-200 patients (16). Such newly available genotyping information is potentially extremely useful to clinicians as they determine warfarin dosage. A recent publication in the Journal of American Medical Association (JAMA) urged clinicians to "establish *CYP2C9* and *VKORC1* genotyping before the first dosing adjustment (usually 3 to 5 days), and to combine pharmacogenomics results with patient physical attributes (weight, age, etc.) to adjust warfarin dosing for particular genotypes" (17). Some examples of this approach based on regression models that incorporated CYP2C9 and/or VKORC1 polymorphisms along with critical demographic and clinical covariates such as gender, weight, and age have been published over last years (18-21).

Consequently, the warfarin (Coumadin®) label has been revised to include genotype indications for dosing and patient safety guidelines, based on genetic variants of the CYP2C9 and VKORC1 genes (www.fda.gov/cder/offices/ODS/MG/warfarinMG.pdf). This FDA-mandated label revision is a landmark event in personalized medicine. The label represents the new paradigm of pharmacogenetic-guided medicine in clinical practice. Notably, this re-labeling relies on meta-analysis of several qualified studies to examine the clinical outcomes associated with *CYP2C9* and/or *VKORC1* gene (especially the -1639G>A allele in the promoter region) variants in stable warfarin-treated patients of Caucasian as well as Asian and African Americans origin. To our understanding, the described relation has not been studied in Hispanics.

The genetic frequencies of the sensitivity and metabolic deficiencies in the Hispanic population are not thoroughly known. There is a clear need for this information. This study is aimed at testing for the effect of common *CYP2C9* and *VKORC1* allelic variants on the optimal maintenance dose of warfarin in a Puerto Rican patient population. If, as expected from studies in other non-Hispanic populations, warfarin dosage differs by genotype, this may support the utilization of genetic factors to predict optimal warfarin maintenance dose in this population at the VACHS main medical center–, but also island wide.

<u>Actions and metabolism of warfarin</u>

Blood coagulation depends in part on the availability of reduced Vitamin K which when oxidized, activates clotting factors II, VII, IX, and X, as well as Proteins C, S, and Z, through gamma carboxylation. The Vitamin K epoxide reductase complex (*VKOR*) reduces Vitamin K, making it available for clotting factors activation. Warfarin exerts its anticoagulant effect through its inhibition of *VKOR*. *VKOR* is coded by the Vitamin K epoxide reductase complex,

subunit 1 (*VKORC1*) gene (22). The Vitamin K epoxide reductase complex subunit-1 (*VKORC1*) is the site of various promoter, exon and intron single nucleotide polymorphisms with resultant highly variable enzyme activity. DNA variants of the *VKORC1* gene alter the sensitivity of *VKOR* to warfarin. About 75% of the Caucasian population carries a DNA variant that reduces expression of the *VKORC1* gene, accounting for 15-27% of clinical variance in warfarin dosage (16).

Warfarin is metabolized to inactive metabolites by the *CYP2C9* isoenzyme of the cytochrome P450 system. The cytochrome P450 enzyme family located in the liver and intestines has evolved to metabolize ingested plant and environmental toxins. The system is responsible for approximately 50% of all drug metabolisms. Genetically deficient *2C9* enzyme polymorphism results in a decreased metabolic clearance of warfarin and resultant drug toxicity with conventional warfarin dosage. Individual differences in drug metabolism influence the variability of drug efficacy and safety leading to a significant impact on clinical management.

Functionally deficient *CYP2C9* variants lead to higher incidence of supra-therapeutic INR values (>4.0), delays in achieving a stable maintenance dose, and increased bleeding complications. *CYP2C9* polymorphisms account for 18-22% of the overall variability in warfarin dosing needs (16).

<u>Non-genetic factors and warfarin dose</u>

Coagulation and warfarin dosing are influenced by multiple factors as follows. Patient age, gender and body size (weight, height) together can enlighten up to 20% of the variance in optimal warfarin dose in American (24) and English (21) Caucasian subjects. Elderly patients often exhibit an enhanced dose response to warfarin, and require lesser dosing. On average, steady-state warfarin doses decrease by 11% per decade of age (25). Physical smaller patients (21) and women also require a lesser warfarin dose (26).

INR is inversely related to dietary intake of Vitamin K (23). In one study, an increase of 714 µg/week of Vitamin K decreased INR by 1.0 unit, equivalent to a 2 mg/day decreased in warfarin dose (23). Evidence suggests that alterations in the dietary intake of vitamin K can affect anticoagulation response to warfarin. It is possible that a low and erratic intake of dietary vitamin K is at least partly responsible for the variable response to warfarin in patients with unstable control of anticoagulation (47). According to an early report by Sconce *et al* (47-48), daily supplementation with oral vitamin K in unstable patients could lead to a more stable anticoagulation response to warfarin. Indeed, these authors found that the mean daily intake of vitamin K in unstable patients was considerably lower than that for stable patients during the study period (29±17µg vs. 76±40µg). In addition, some medications alter the anticoagulant effect of warfarin. Many compete with warfarin for metabolism through the

CYP2C9 system. Such medications would introduce different degrees of change in the warfarin dose for those with *CYP2C9**2, or *3 allele variants (16).

Warfarin therapy: trial and error

Initiation of warfarin anticoagulant therapy is specially challenging because the therapeutic dose is highly variable. Warfarin effectiveness is gauged by the INR, which is a measure of anticoagulation adequacy. To attain the appropriate INR therapeutic range decreases the risk of bleeding. INR therapeutic range is 2.0-3.0 for most anticoagulation indications (2), although higher INR therapeutic range (2.5 – 3.5) is indicated for patients with heart valve replacement (mitral/aortic) and hypercoagulable state disease. Warfarin therapeutic doses usually range from 0.5 to 10 mg/day (2). Titration of warfarin dose in a new patient is accomplished by the prescription of an initial dose and then, adjusting the dose according to close INR monitoring. An INR under target range, due to a low warfarin dose, may lead to increased risk for thromboembolic events. On the other hand, an INR over target range due to a high warfarin dose can result in over-anticoagulation and increased risk for bleeding complications. A slow cautious approach averts bleeding complications but the process is often expensive, time-consuming (may require weeks), and may fail to provide the needed therapeutic benefit in a timely fashion.

Quantifying the genetic component to warfarin sensitivity

Warfarin (Coumadin®) has been available for approximately 50 years, but recently the considerable genetic influences on its effectiveness have become known to the scientific community. The *CYP2C9* gene is of great relevance in cardiovascular medicine as its product metabolizes warfarin. *VKORC1* is recognized by its role in facilitation of clotting. Major population differences account for clinical variance in anticoagulation efficacy and safety. Wadelius and Pirmohamed (16) noted that warfarin sensitivity in English (21), Swedish (14), Italian (27), Japanese (28), and other Asian populations is significantly affected by variation in at least two genes, *VKORC1* and *CYP2C9*.

The proportion of inter-individual variance in the INR attributable to combinations of the gene variants varies according to ethnicity and perhaps the continent of origin. The proportion of variance in warfarin dose explained by the *CYP2C9* and *VKORC1* genotype variants in combination is 32-49%, based on data in English (32%)(21), Swedish (40%) (14), American Caucasian (49%) (24) and Hong Kong Chinese (39%) (29). There are no published data indicating allelic frequencies of CYP2C9 or VKORC1 alleles in Hispanic populations and very limited data on small samples of African Americans.

<u>INR stabilization in chronic therapy with warfarin</u>

It is estimated that patients on chronic warfarin therapy are on average within their target range for only two-thirds of the time (50). As a consequence, they are at risk of hemorrhage and thromboembolism, resulting from over- and under- anticoagulation, respectively (51). Although several factors including age, concurrent medication, co-morbidity and compliance are known to affect anticoagulation in a predictable way, at present a large part of intra-individual variation in warfarin response cannot be explained (47-48).

<u>Relevance to the VA Caribbean Healthcare System (VACHS) population</u>

The VACHS main medical center in San Juan, Puerto Rico currently prescribes warfarin to approximately 1640 patients. The determination and maintenance of the optimal warfarin dose for a given individual can require many clinic visits and frequent INR testing to adjust warfarin dose. There are several medical issues inherent to the management of warfarin therapy that might be life-threatening and can potentially be improved by taking DNA typing in consideration. The number of clinic visits needed to achieve therapeutic INR range could be reduced as well as the number of dosage adjustments due to change in concurrent CYP2C9 substrate drugs, the time to achieve therapeutic warfarin levels in a new patient, the number of INR test ordered, the number of bleeding complications, the number of INR test results above 4.0 or below 2.0 and the clinician's time spent to manage warfarin therapy. The complexity associated with co-morbid conditions often makes the care of the anti-coagulated patient difficult and troublesome for the practicing clinicians.

Rationale

A gap exists in the literature with respect to DNA typing of persons of Hispanic ancestry in relation to warfarin sensitivity. The current approach to prescription of initial and maintenance warfarin dose is empiric, lacks a genetic component, and often results in bleeding complications requiring hospitalization despite careful monitoring with PT and INR (2). This is a first step in the establishment of genomic-based warfarin therapy in the Puerto Rican (Hispanic) population. Ultimately, prediction of maintenance dose based on DNA typing will permit more efficient individualization of warfarin dose leading to improved patient safety and decreased complication-related expenses. Widespread integration of genomic based warfarin therapy could help warfarin users to avoid up to 17,000 strokes, 43,000 adverse drug effects, and 85,000 serious bleeding episodes annually (2). This research could advance DNA-guided medicine to alleviate health care disparities and the economic benefits will also be significant.

On the other hand, to date there are still limited published data providing information about the relation of dietary intake of vitamin K to anticoagulation response to warfarin in patients

with unstable control of anticoagulation and how polymorphisms in the *VKORC1* gene are associated with such vitamin K intake-related unstable control. No such data have been published as yet for patients of Hispanic Puerto Rican origin. This Pharmacogenetic and dietary approach can then be recommended as the basis for the VACHS in Puerto Rico (and perhaps to Puerto Rico) quality improvement initiative of patient outcomes and reduction of adverse clinical events after warfarin administration.

Significance to the Hospital: The study will provide the VACHS clinicians with new and essential genetic and dietary information that is expected to make a more predictable management of warfarin therapy, enhance treatment effectiveness, and decrease warfarin therapy complications for patients and clinicians. Both will benefit from decreased time needed to attain the therapeutic warfarin dose and a decreased number of: 1)dose adjustments; 2) clinic visits needed to arrive at the therapeutic dose; 3) INR tests ordered; 4) out of therapeutic range INRs [>4.0 or <2.0]; 5) bleeding complications. The VACHS medical center will benefit from a reduction in the length of stay from these patients for the reasons outlined above and will also benefit medically by providing more personalized health care.

In the short term, this proposal will generate high impact peer reviewed publications and be leveraged with foundation and NIH grant applications. Since our algorithms will be developed in a Hispanic population to address their primary care needs with novel DNA-guided technology, we believe the results could form a forerunner of DNA-guided medicine to address healthcare disparities. Warfarin has emerged as one of the best cases for personalized medicine in real clinical practice. The National Heart Lung and Blood Institute will be sponsoring a prospective genotype-enhanced warfarin protocol at a national scale. Success with our proposal can position the VACHS and UPR-MSC to compete in this new NHLBI initiative. In addition, this grant would aid the positioning of the VACHS medical center as a leader in patient safety. On October 6, 2006, Bristol Myers Squib added a new black box warning that warfarin can cause mayor fatal bleeding (http//www.fda.gov/MEDWATCH/SAFETY/2006/safety06.htm-Coumadin). A new patient medication guide will accompany all warfarin prescriptions. Such guide indicates that the FDA has determined that warfarin poses a serious and significant public health concern.

Finally, the study will lay the groundwork for personalized anti-coagulant therapy, bringing pharmacogenomics to real clinical environment and complementing genome-based, research efforts of faculty in the School of Pharmacy, University of Puerto Rico. This study will allow us to initiate personalized medicine at the UPR-MSC and the VACHCS inserting our efforts in the pioneering role of clinical pharmacogenetics research in Puerto Rico.

Hypotheses

Primary Hypothesis:

Question 1: What are the allele frequencies of the *CYP2C9, CYP4F2, GGCX, NQO1, EPHX1* and *VKORC1* genes in a Hispanic (Puerto Rican) ambulatory population and how do they compare to other populations?

Hypothesis 1: The allele frequencies of the *CYP2C9, CYP4F2, GGCX, NQO1, EPHX1* and *VKORC1* genes in the Hispanic (Puerto Rican) population will differ from other populations due to its admixed and heterogeneous ethno-geographic heritage.

Secondary Hypothesis:

Question 2: Will warfarin maintenance doses (MD) and the anticoagulation-related outcomes differ in Puerto Rican patients who carry *CYP2C9, CYP4F2, GGCX, NQO1, EPHX1* and *VKORC1* gene allele variants?

Hypothesis 2A: Carriers of *CYP2C9* and/or *VKORC1* and/or *GGCX* and/or *EPHX1* polymorphisms will demonstrate lower MD of warfarin compared to those homozygous for the wild type genotype.

Hypothesis 2B: Carriers of *CYP4F2* and/or *NQO1* and/or *VKORC1* variants will demonstrate higher MD of warfarin compared to wild type alleles.

Hypothesis 2C: Carriers of *CYP2C9* and/or *CYP4F2* and/or *GGCX* and/or *NQO1* and/or *EPHX1* and/or *VKORC1* polymorphisms in the Puerto Rican population will demonstrate higher over-anticoagulation risk, higher rate of out-of-range INRs, higher incidence of serious or life-threatening bleeding events, thromboembolism or strokes and longer time to target therapeutic INR and stable warfarin dosing as compared to wild-type.

Question 3: Will simultaneous genetic determination of the *CYP2C9, CYP4F2, GGCX, NQO1, EPHX1* and *VKORC1* genetic variants reliably predict the therapeutic dose of warfarin?

Hypothesis 3: *CYP2C9, CYP4F2, GGCX, NQO1, EPHX1* and *VKORC1* genetic variants will significantly predict the optimal therapeutic dose of warfarin, i.e., that needed to produce an INR in the range of 2.0 to 3.0 in the VACHS population.

Question 4: Does the low intake of dietary vitamin K contribute to a larger intra-individual variability in anticoagulation response to warfarin in unstable Puerto Rican patients of the VACHS-affiliated anticoagulation clinic, which is more likely in patients who also carry a *VKORC1* polymorphism?

Hypothesis 4A: A low and inconsistent intake of dietary vitamin K will contributes to intra-individual variability in anticoagulation response, with those with unstable control of anticoagulation having a lower or more variable intake than those with stable control.

Hypothesis 4B: Unstable anticoagulation control due to low intake of dietary vitamin K is more likely to happen and more severe in those carriers of the *VKORC1*-1639G>A variant.

Question 5: Is the pharmacogenetic testing of *CYP2C9* and *VKORC1* genetic variants a cost-effective method in the clinical management of high risk warfarin-treated patients during the induction phase at the VACHS-affiliated anticoagulation clinic?
Hypothesis 5: The pharmacogenetic testing of high risk warfarin-treated patients at the VACHS-affiliated anticoagulation clinic is more cost-effective than the routine INR monitoring to control anticoagulation during initial phase.

Objectives

1. To determine prevalence (allele and haplotype frequency distributions) of *CYP2C9*, *CYP4F2, GGCX, NQO1, EPHX1* and *VKORC1* polymorphisms in Puerto Rican patients and to compare them with other early reported populations.
2. To determine whether combinatorial *CYP2C9*, *CYP4F2, GGCX, NQO1, EPHX1*, *VKORC1* genotypes are associated with clinical phenotypes and may explain variability in stable warfarin maintenance dosage in a Puerto Rican population.
3. To develop a DNA-guided warfarin dosing algorithm in the Puerto Rican population.
4. To evaluate the relationship of dietary intake of vitamin K to the anticoagulation response to warfarin in unstable Puerto Rican patients compared to those with stable control of anticoagulation.
5. To determine whether the *VKORC1*-1639 G>A polymorphism is either a confounder or an effect modifier of the dietary relationship to the anticoagulation status in Puerto Rican patients at VACHS.
6. To determine economic (cost-effectiveness) viability of DNA-testing in the VACHS-affiliated anticoagulation clinic through a decision analysis modeling of the time and costs of genotyping in warfarin-treated Puerto Ricans with high risk of adverse reactions.

Methodology

A. Study Design

This is an open label, retrospective, single-center pharmacogenetic study (genotype – phenotype association) in Puerto Rican patients on stable warfarin therapy. The distribution frequencies of the clinically relevant *CYP2C9, CYP4F2, GGCX, NQO1, EPHX1* and *VKORC1* allelic variants in the study cohort will be tested by a cross-sectional (snapshot) prevalence study. Comparisons will be performed versus earlier published data from other populations. This clinical trial protocol will be submitted to the Institutional Review Board (IRB) of both the VACHS and the UPR-MSC for approval. Subjects enrolled in the trial must

be able to give informed consent prior to accrual. A waiver for authorization to access medical records of potential candidates will be requested in order to identify those individuals who meet inclusion criteria. Eligible candidates, who meet enrollment criteria, will be first informed about the study by his/her primary healthcare provider (PharmD or clinicians from the VACHS anticoagulation clinic), asked about his/her interest to participate and, if agree, interviewed by the PI and study personnel at the time of his/her next scheduled appointment. They will then be invited to participate in the study and be asked to complete a questionnaire consisting in multiple-choice questions about his/her routine consumption vitamin K-rich foods and beverages (see appendix A). The PIs will be responsible to provide each potential candidate with the corresponding orientation regarding his/her potential participation in the study and assurance of patient understanding of the study and participation risks-benefits. A small blood sample (about 5ml) for DNA analysis will be taken from each participant at the same time of his/her next routinely INR measurement in the corresponding VACHS facilities. This procedure will be performed by well-experienced phlebotomist of the VACHS in order to minimize potential risk upon blood withdrawal. No additional appointments and/or specimen collections will be scheduled. Blood samples in the containers will be fractionated into two to three portions for retrieval and replicate purposes. The sample collected in vacutainers from each patient will be immediately coded by the study coordinator using a seven-digit unique study number printed on each specimen tube label so that the sample will not directly identify the patient. No other labels or identification will be printed on the sample container. Containers will then be placed on ice and stored frozen until genomic DNA extractions at the Molecular Genetics Laboratory (A-640, 6th floor, Main Building, University of Puerto Rico, Medical Sciences Campus). Each extracted DNA sample will also be identified using another seven-digit unique study number printed on the corresponding tube label and frozen until assay. DNA samples will then be tested for genotyping (Genomas-Laboratory of Personalized Health, Hartford Hospital, CT). Demographic and non-genetic data will be retrospectively recorded from computerized patient record system (CPRS) and questionnaire.

To determine whether combinatorial *CYP2C9*, *CYP4F2, GGCX, NQO1, EPHX1* and *VKORC1* genotypes are associated with clinical phenotypes in this study population, survival analysis techniques (Kaplan-Meier time-to-event analysis for anticoagulation-related outcomes and bleeding episodes) will be used. To determine whether the major *CYP2C9*, *CYP4F2, GGCX, NQO1, EPHX1* and *VKORC1* allele variants explain variability in stable warfarin dosage in this study population, a multiple linear regression analysis will be performed using maintenance warfarin dose as the dependent variable and following a stepwise regression procedures. Based on their partial correlations after considering the effect of genotypes, we will also consider age, body size, sex, admixture (ancestry), concomitant medications, indications and Vitamin K intake as potential regressor variables that independently explain warfarin dose variation. Full explanation for regression modeling

is provided in the statistical section of this protocol (see hypothesis 3 for details). Using this regression model a warfarin-dosing algorithm for the VACHS Puerto Rican population that predicts the best dose for stable anticoagulation will be developed.

The study is expected to take a period of three to four years. There will be no changes to patient's treatment and/or dosing regimens or genetic counseling based on the results of his/her DNA test. Graduate and Doctor of Pharmacy students will assist in some of the scheduled activities (data entry, assembly, data analysis), except in those tasks requiring specialized skills by certified healthcare personnel and/or involving personal (individual) identifiable data. PIs at the VACHS will be responsible for subject recruitment, obtaining informed consent and collection of demographic and clinically relevant data. Co-PI at UPR-School of Pharmacy will be responsible for assays, data analysis, reports and result interpretations.

Data Confidentiality and Privacy

Proper safeguards against any potential violation of privacy and/or breach of confidentiality will be provided. Any relevant patient's information that can be considered as identifiers will be kept safely by the PIs of the study in encrypted and separated patient's records apart from the study database and codes. All these records will electronically reside in the VA server (VACHS, San Juan facilities) as well as the files containing the codes for both blood and extracted DNA samples, which link personal identifiable information with the relevant research-related data obtained from the study. Codes will also be stored encrypted in separate files from records and databases. Codes will only be used to be able to perform the necessary data assembly. It will be done by making each individual unique genotype (i.e., wild-type, single carrier, double carrier) and his/her related demographic/ clinical covariates (e.g., weight, sex, gender) correspond to his/her stable warfarin dose and/or anticoagulation-related outcomes as specified in the protocol procedures. In doing so, we will be able to undertake the statistical analysis in an attempt to identify whether clinical correlations exist. Codes will neither contain nor scramble letters/numbers that might be linked to individually identifiable information from patients enrolled in this study. After completing the protocol, the VA employee who is the study PI will store these master records/files, study databases and any other relevant document related to this study and its findings within the VACHS Research Office. Any other file containing relevant data collected from VACHS records and used to perform any specialized analysis outside the VACHS facility will be deleted following an accepted de-identification procedure (overwriting, degaussing, etc). Once this is done there will be no way to know which specific sample the patient provided. All the specimens (ie, patients' blood samples and extracted DNAs; including fractions for retrieval and/or replications) that we process as part of this study will be used for only the specific purpose defined in this protocol and will be destroyed at the end of the project. In doing so, the risk of any privacy violation will be controlled. Individual blood samples will be completely discarded

at the moment of DNA extraction, following the current policy at UPR-Medical Sciences Campus for safety disposal of biohazard materials. De-identified DNA specimens will be stored frozen in eppendorf tubes at the UPR-Medical Sciences Campus facility (Co-PI's Lab A-640, RCMI Core Lab of Molecular Genetics, 6th floor, main building) until the end of the study. To this purpose, a 7-digit code will be used to link each DNA specimen with the corresponding personal identifiable information that will only reside on VA protected environment at the PI's office.

Collaborators in the study, and particularly the personnel at either the Laboratory of Personalized Health-Genetic Research Center (Hartford, CT) or the RCMI Molecular Genetic Core Lab (Medical Sciences Campus, UPR), will not have access to any information that identify the enrolled patients. All specimens will be destroyed and properly disposed after processed therein. Caution will be observed to protect and avoid unnecessary disclosure of any research-related health information arisen during this study because such action will be irrelevant for the purposes of this survey. The protected health information will not be reused or disclosed to any other person or entity. The study data might be reviewed by the ethics committee overseeing the research or the VACHS to monitor the study. The study data may also be reviewed by regulatory authorities.

Personal (individual) identifiable information and/or any other relevant research-related health data from candidates will only be collected, assembled and accessed by the research team members of this study protocol. The PIs and/or study coordinator will be responsible for overseeing the security and confidentiality of such records. This information will mainly be copied from patients' medical records using the computerized patient record system (CPRS) after obtaining in advance the corresponding signed authorizations from patients. Demographic and other clinical data of interest (e.g., height, weight, age, sex, co-morbidities, concomitant medications, etc) that will be collected from clinical records, as well as dietary Vitamin K intake (questionnaire) and combinatorial genotyping data, will be entered into a MS Excel worksheet, assembled by matching up all data/information collected from each individual subject and then saved as a password-protected .xls file in a VA's proprietary laptop. PI and Co-PI will be the holders of the laptop after receiving proper authorization. If needed for further statistical analysis, data (excluding patient name, medical record, or any other identifier that would reveal patient identity) can be exported using encrypted language to secure information against eventual lost or theft. Raw data from genotyping analysis of each sample as well as details of the performed procedures/assays to call a genotype will be recorded on the corresponding work books, which resides within the laboratory facilities, as required by good laboratory practice and UPR-MSC policies. However, such raw data are totally de-identified and, therefore, there are no risk of privacy or confidentiality breach. Removal of access to research study data will be accomplished for study personnel when they are no longer part of the research team.

Information to be used and/or disclosed for research purposes

The following *Protected Health Information* (PHI) and/or *Individually Identifiable Information* (III) will be collected from recruited patients to be used and/or disclosed in order to achieve the research purposes of this study, as described above: a) information provided directly by the participant to the research team (e.g., by questionnaires); b) information collected directly from the computerized patient record system (CPRS) by the research team. Such information includes history and physical examination; diagnostic/laboratory test results; prescriptions; consultations; and clinic and progress notes. Although the PI (Dr. Giselle Rivera) and her research team will be the only ones allowed to use this information, they may share some patient's PHI with the following parties in order to audit or monitor the quality and safety of the research activity: Institutional Review Boards, the Department of Health & Human Services or other US government agencies, as required by law. To this purpose, every patient who consents to participate in this study will also sign a HIPAA authorization form that will be valid until the end of the study.

Data Safety Monitoring Plan (DSMP)

The study is designed to evaluate implementation feasibility and initial appropriateness of a DNA-guided warfarin dosing algorithm by means of genotypic information collection and analysis. Participants will be recruited from the VACHS Anticoagulation Clinic. At the conclusion of the protocol, we expect that the relevance and usefulness of obtained data will justify the evaluation of such analysis on a larger scale.

1. Potential undesired events resulting from participation includes:

- possible violation of confidentiality,
- possible discomfort due to assessment procedures,
- possible dissatisfaction with the assessment procedures

2. Procedures to safeguard against undesired events:

All data collection protocols include a form on which research staff members record any problems with the data collection, concerns about the patient, or unusual occurrences during the blood sample or data collection. These forms allow our research staff an opportunity to quickly review and respond to any possible concern or undesired event.

2.1. Informed Consent:

All participants are required to read and sign detailed assent/consent forms before participating in the study. Participants are advised of the voluntary nature of participation and

of their right to withdraw from the project at any time and to require that information about them be removed from data analysis. Each study participant receives a verbal and written description of the study. Experienced research staff members are available at all points of the study process to answer questions and to explain assessment procedures, uses to which the data will be put, and confidentiality of data. As our population includes only Hispanic patients, all project documents, including the consent/assent forms, will be available in both Spanish and English. If a staff member determines that a participant's reading skills are limited, all materials will be presented verbally in Spanish or in English, as indicated and questionnaires will be administered as an interview.

2.2. Confidentiality Safeguards:

To ensure confidentiality, all research-derived VA sensitive information will be coded so that it cannot be associated with any individual. Individual names, addresses, social security numbers and/or any other identifiers are not necessary for the protocol performance instead their blood samples that will be identified by code numbers. All data entered into the computerized database will be identified by subject’s code number only. The personal information will be maintained on a VA server and documentation of the procedure and the code key by which the data are coded will remain within the authorized VA facilities. All original VA sensitive research information will reside on a VA server. Any original electronic VA research data will be backed up regularly and stored securely within VA’s protected environment. All Center staff and University staff sign confidentiality agreements and have completed the appropriate VA data security training. No one will have access to records identifying subjects' names and/or other identifiers at any time. All coded data sent outside the VA facilities will be encrypted and have tracking documents attached to ensure “chain of custody”. The information gathered will be used only for VA-approved scientific, educational or instructional purposes. The study personnel who have access to, use, store, or transport the data are familiar with the VA policy and facilities requirements for reporting theft or loss of sensitive data or the media (VA issued and encrypted flash drives) containing sensitive data. In accordance with VA policy, procedures will be in place for reporting incidents such as theft or loss of data or storage media, unauthorized access of sensitive data or storage devices or non-compliance with security controls.

2.3. Staff Training Safeguards:

Every research staff member is required to participate in a mandatory, ongoing training program on human subject protection. Key personnel are required to complete a more extensive, three-part training/testing program. Research staff receive training specific to each assessment tool. All project staff meets on a weekly basis for ongoing training, monitoring of protocol, and problem solving. Questions regarding data collection are promptly referred to the PI.

2.4. Data Safeguards:

Access to Center buildings is restricted. Reception staff members supervise public entrances during normal operating hours. All space accessible to the public is separated from research offices by locked, coded doors. All staff entrances are secured with locked, coded doors. Staff entry codes are changed regularly.

The computing system which gathers the VA research data relevant to this study (PC located in the PI's office room #1E-129; building OPA) is protected from outside access by a two-tiered firewall system: the Linux Server running Net filter is backed-up by the Windows Server running Microsoft Internet Security and Acceleration Server (ISA). The Net filter and ISA firewall software allows us to define rules that block any attempt to compromise the system. Both servers maintain logs and generate reports of access attempts, which are reviewed by the Network Administrator.

During the analysis of the data, all identifying information with the exception of the subject identification number is removed from the data. No information about the identities of study participants will be published or presented at conferences.

2.5. Discomfort with Assessment Procedures or Disclosure Safeguards:

During the course of participation in the research, a participant may have questions about the assessment procedure. A project staff member will be available to answer questions.

3. Response procedures for undesired events:

During initial training, staff members are coached to respond to embarrassment or discomfort in an appropriate and compassionate manner. Participants will also be encouraged to contact the project PI and other project staff in the event of doubts about project procedures, safeguards or finalities.

4. Oversight:

The research staff is responsible for the general oversight for all project activities and will inform the UPR and VACHS about changes and requirements for the DSMP. The research staff will update the general DSMP procedures as needed.

B. Sample

We plan to recruit 300 patients over a 3-4 years total period. The targeted 300 patient study population cohort will only include patients clinically followed at the Anticoagulation clinic of

the VACHS, who are currently receiving therapeutic doses of warfarin. The sample size is calculated according to procedures detailed on the statistical section of the study.

C. Enrollment Criteria

Inclusion Criteria

- Age ≥21, but <90 years old
- Stable warfarin dose –three consecutive INRs within therapeutic range (2 – 3 or 2.5 – 3.5, according to indication) for the same dose
- Receiving warfarin for therapeutic anti-coagulation in indications such as deep vein thrombosis (DVT) with or without Pulmonary Embolism (PE); atrial fibrillation or other arrhythmias; cardiac valvular replacement; previously diagnosed coagulopathy
- Hct > 40%

For the accomplishment of ***Objectives #4 and 5*** (Amendment #3)

- Patients with unstable INR values (defined as patients with standard deviation >0.5 in INR values and has at least three dose changes during the previous six months).
- Age ≥21, but <90 years old
- Receiving warfarin for therapeutic anti-coagulation in indications such as deep vein thrombosis (DVT) with or without Pulmonary Embolism (PE); atrial fibrillation or other arrhythmias; cardiac valvular replacement; previously diagnosed coagulopathy
- Hct > 40%

Exclusion Criteria

- Non-Hispanic patients (race/ethnicity is self-reported by the patients)
- Currently enrolled in another active research protocol at the VACHS Hospital
- BUN/creatinine >30/2.0 mg/dL
- Active hepatic disease (defined by a Child-Pugh score above 10 points: ascites; total bilirubin above 2.0 mg/dl; serum albumin below 3.5 g/dl; prothrombin time in seconds prolonged over control >4; hepatic encephalopathy)
- Prolonged diarrhea (three or more days)
- Nasogastric or enteral feedings
- Acute illness (e.g., sepsis, infection, anemia)
- HIV/AIDS, Hepatitis B patients
- Alcoholism and drug abuse
- Patients with any cognitive and mental health impairment

LFT <3x ULN
Sickle cell patients
Active malignancy

For the accomplishment of ***Objectives #4 and 5*** (Amendment #3)

Patients with well-documented instabilities due to poor compliance, concurrent medication, illness or irregular and excessive alcohol consumption
Non-Hispanic patients (race/ethnicity is self-reported by the patients)
Currently enrolled in another active research protocols at the VACHS Hospital
BUN/creatinine >30/2.0 mg/dL
Active hepatic disease (defined by a Child-Pugh score above 10 points: ascites; total bilirubin above 2.0 mg/dl; serum albumin below 3.5 g/dl; prothrombin time in seconds prolonged over control >4; hepatic encephalopathy)
Prolonged diarrhea (three or more days)
Nasogastric or enteral feedings
Acute illness (e.g., sepsis, infection, anemia)
LFT <3x ULN
Active malignancy

D. Data Collection

<u>Variables to be collected</u>

Genotypes: The most common alleles of the *CYP2C9* gene (*1, *2, *3) responsible for the metabolism of warfarin, and two most common alleles for the *VKORC1* gene (G/A at -1639) responsible for the action of warfarin, will be the focus of this study (see **Table 1** below).

Table 1 *CYP2C9* and *VKORC1* alleles, their frequencies and phenotypic effects in Caucasians.

CYP2C9					VKORC1				
**Alleles**	**DNA variant**	**Change to Protein**	**Activity**	**Freq.**	**Alleles**	**DNA variant**	**Change to Protein**	**Activity**	**Freq.**
*1	Wild Type	Reference	Normal	86%	WT	Wild Type	Reference	Normal	57%
*2	430C>T	Arg144 Cys	Decr.	9%	-1639	G>A	Promoter	Deficient	43%
*3	1075A>C	Ile359Leu	~Null	4%	+85	G>T	Val29Leu	~Null	Rare
*4	1076T>C	Ile359Tyr	Decr.	1% Far East	+121	G>T	Ala41Ser	~Null	Rare
*5	1080C>G	Asp360Glu	Decr.	African-American	+134	T>C	Val45Ala	~Null	Rare
*6	818delA	Frameshift	~Null	African-American	+172	A>G	Arg58Gly	~Null	Rare
					+1331	G>A	Val66Met	~Null	Rare
					+3487	T>G	Leu128Arg	~Null	Rare

However, our laboratory analysis will also include other important but less common or missed (unknown) allele variants in these loci, as well as polymorphisms in the following additional candidate genes: *CYP4F2, GGCX, NQO1, and EPHX1*, which have been recently postulated to be also involved in warfarin's response variability (see **Table 2** below). Should the less common or unknown alleles, or any of the polymorphisms in the newly candidate genes, occur more frequently in the predominantly Hispanic population to be studied, we will utilize them in our predictive approach. Genetic ancestry (admixture) will be determined based on the physiogenomic analysis of each individual.

Table 2 *CYP4F2, EPHX1* and *GGCX* alleles to be tested, their frequencies in Caucasians and expected phenotypic effects.

Gene	SNP	DNA variant	Change to Protein	Activity	Freq.
**CYP4F2**	rs2108622	C>T	Val>Met (433)	Resistant	22-29%
**EPHX1**	rs1051740	C>T	Tyr>His (113)	Deficient	28-35%
	rs2292566	A>G	Lys>Lys	Deficient	Rare
	rs2260863	C>G	Intron 3	Deficient	Rare
	rs2234922	A>G	His>Arg (139)	Deficient	Rare
	rs4653436	A>G	Upstream	Deficient	Rare
**GGCX**	rs12714145	C>T	Intron 2	Deficient	26-31%
**NQO1**	rs1800566	C>T	Pro>Ser (187)	Resistant	Rare

Other Variables: Demographic data including age, gender, height, weight, admixture, self-reported racial/ethnicity and other clinical data including dietary intake of Vitamin K, concurrent medications, stable warfarin dose (mg/day), target INR range, INR values, bleeding complications, indication for warfarin therapy, and concomitant diseases will also be collected from medical records and questionnaire.

Main Outcome Measures (time to event): The primary end-point will be the *anticoagulation status*, as measured by INR in three ways: time to therapeutic INR, time to first above-range INR and time to stable warfarin dosing (defined as three consecutive clinic visits for which INR measurements are within therapeutic range for the same mean daily dose); the second end-point is time until first severe or life-threatening bleeding events. Once patients achieve stable dosing, the maintenance warfarin dose will be recorded in order to compare mean maintenance dose among the various genotypes. Therapeutic INR is defined as the first measured within the optimal therapeutic range for a given indication. If the target therapeutic INR range is 2.0-3.0, then INRs between 2.0 and 3.0 will be defined as within range. Above-range INRs will be defined as measurements of 4.0 or greater. Patients receiving warfarin for prosthetic valve replacement are usually anticoagulated at a higher target range (2.5-3.5). Consequently, these patients will require an above-range INR definition of 4.5 or greater to account for their higher baseline level of anticoagulation (31). This is to ensure these patients will not contribute disproportionately to the analysis. The minimum cutoff for above-range INR is considered to be 4.0 because INR measurements above this level are less likely to be

misclassified as above-range when compared with measurements below 4.0. INR values between 3.0 and 4.0 are not considered to be strong predictors of bleeding risk (31). 4.0 or greater have been previously used to define “above range” (31). We will follow the same criteria used by Higashi et al (31), based on Fihn’s protocol, to classify bleeding episodes as serious (requiring treatment or medical evaluation) or as life threatening. Examples of serious bleeding events include overt gastrointestinal bleeding, occult gastrointestinal bleeding if endoscopic or radiographic studies are performed, gross hematuria that prompt cystoscopy or intravenous urography or last more than two days, and hemoptysis. Episodes involving blood transfusions of two units or more will be classified as serious bleeding events. Life threatening bleedings are those leading to cardiopulmonary arrest, surgical or angiographic intervention, or irreversible sequelae such as myocardial infarction, neurological deficit consequent to intra-cerebral hemorrhage or massive hemothorax. Bleeding is also considered to be life threatening if result in two of the following consequences: loss of three or more units of blood, systolic hypotension (<90 mmHg) or critical anemia (hematocrit of 20% or less).

See **appendix B** for data collection form copy.

Methods of Data Collection

Demographic and clinic data: All relevant non-genetic data will be retrospectively obtained from computerized patient record system (CPRS) and questionnaire. These will be recorded on a MS Excel formatted clinical database by the PIs.

Blood collection for DNA typing: 5 ml EDTA blood sample per patient will be drawn in lavender/purple-colored stopper vacutainers tube for genotyping, at the time of routinely scheduled PT/INR testing collections. The tube will be gently inverted approximately 10 times to insure proper mixing. The corresponding patient’s code number will be printed on the tube label.

Sample Preparation and DNA Quantification

Genomic DNA samples will be extracted from whole blood using QIAamp DNA Blood Midi Kit (Qiagen, Valencia, CA) following manufacturers protocol. Extracted DNA will be stored at -80° in TRIS-EDTA buffer (TE). Quantification of DNA will be performed by fluorescent staining of double stranded DNA (PicoGreen® dsDNA Quantitation Kit, Molecular Probes, Eugene, OR). Fluorescent intensity will be measured using a fluorescent micro-titer plate reader (POLARstar OPTIMA, BMG LABTECH GmbH, Offenburg, Germany). The concentration of extracted DNA will be adjusted to 12.5 ng/μL, in DNase free distilled water. Around 25-50 ng of extracted DNA will be required for performing the genotyping assay (i.e.,

Tag-It™ Mutation Detection Kits; TaqMan SNP assay; sequencing; Affymetrix DMET chip assay and/or Illumina-based physiogenomic (PG) array).

DNA typing assays

Luminex Assay: DNA typing by Luminex assays will be performed at the laboratory of Personalized Health (LPH), using the HILOmet Warfarin system. The LPH is located at the Hartford Hospital Genetics Research Center in The Florence Crane Building, 67 Jefferson Street (Hartford, CT) and has been in operation since April 2005. LPH is a high complexity clinical DNA testing center licensed by the Connecticut Department of Health (CL-0644) and certified by the Centers for Medicare and Medicaid Services (ID# 07D1036625) under CLIA (Clinical Laboratory Improvement Amendments). The HILOmet system employs a Luminex® 100 analyzer using xMAP® technology (Luminex Corp., Austin, TX) installed at the LPH. The genotyping kits are from Tm Bioscience (Toronto, Ontario, Canada). The assay requires 50 ng genomic DNA.

The Tag-It™ Mutation Detection Assays (TM BioSciences, Toronto, Canada) will be utilized for DNA typing the *CYP2C9* and *VKORC1* genes at 12 variables sites, 5 alleles in *CYP2C9* and 7 alleles in *VKORC1* (see Table 1). These assays employed PCR to amplify selectively the desired genes without co-amplifying pseudogenes or other closely related sequences (30). The kits use multiplexed Allele Specific Primer Extension (ASPE) to identify small nucleotide variations including single base changes and deletions of one or three bases. In brief, a PCR-derived target DNA wit two universally-tagged allele-specific primers whose 3' ends define the alleles was used for each variation tested. A thermophilic DNA polymerase is used for primer extension and biotin-dCTP Label Corporation. Because the two tagged allele-specific primers overlap the SNP site in the target DNA, only the correctly hybridized primers was extended to generate labeled products. Single tagged ASPE primers are used to detect the presence of unique PCR fragments generated for the deletion and duplication gene rearrangements. Following ASPE, tagged, extended products labeled with biotin were captured by their tag complements (anti-tags), which had been chemically coupled to spectrally addressable polystyrene microspheres. A fluorescent reporter molecule (streptavidin-phycoerythrin) was used to detect incorporated biotin. The fluorescent reporter signals generated for each bead population was measured on the Luminex xMAP™ system (Luminex Corporation, Austin, TX).

Conventions for naming the alleles according to the Human Cytochrome P450 (CYP) Allele Nomenclature Committee will be followed in this research (http://www.imm.ki.se/CYPalleles/). Accordingly, all alleles contain nucleotide changes that have been shown to affect transcription, splicing, translation, posttranscriptional or posttranslational modifications or result at least one amino acid change. VKORC1 alleles are named according to their site in the gene, based on nucleotide number.

DNA typing by Luminex and Tm Bioscience Tag-It Analyte Specific Reagents: The Luminex xMAP technology is based on 100 distinct sets of color-coded polystyrene beads with diameter of 5'6 μm, called microspheres. Each bead set can be coated with a reagent specific to a particular bioassay, allowing the capture and detection of specific analytes from a sample. Within the Luminex 100 compact analyzer, lasers excite the internal dyes that identify each microsphere particle, and also any reporter dye captured during the assay. Many readings are made on each bead set, further within a single sample, both rapidly and precisely.

Tm Bioscience is a DNA-based diagnostics company developing suite of genetic tests, including bioassays for genetic mutations or SNPs related variants of cytochrome P450 genes. The test kits are Analyte Specific Reagents (ASRs). Tm Bioscience is currently applying at the Food and Drug Administration for 510(k) approval of their Tag-It™ Mutation Detection Kits. The Tag-It™ Mutation Detection Kits follow a four step process:

Multiple PCR,
Multiplex Allele-Specific Primer Extension,
Universal Array Sorting,
xMAP Detection.

Multiplex PCR: The Tag-It ™ assays employ PCR to selectively amplify the target gene without co-amplifying any of the closely related pseudogenes. In addition, PCR is used to amplify genomic fragments characteristic of the deletion and duplication genotypes.

Multiplex Allele-Specific Primer Extension (ASPE): ASPE chemistry is a simple, robust method for analyzing multiple variations in a single tube. For each variation to be tested, the method employs a PCR-derived target DNA with two universally-tagged allele-specific primers whose 3'ends define the alleles. A thermophilic DNA polymerase polymerase is used for primer extension and biotin-dCTP label incorporation. Because the two tagged allele-specific primers overlap the SNP site in the target DNA, only the correctly hybridized primers will be extended to generate labeled products. A non-complementary primer will be neither extended nor labeled due to the 3'mismatched base. ASPE is also used to detect the presence of unique PCR fragments generated for the deletion and duplication gene rearrangements.

Universal Array Sorting: Following ASPE, aliquots of the reaction are added directly to microwells containing bead-immobilized anti-tags. Tagged, extended products labeled with biotin during ASPE are captured by their tag complements (anti-tags) which have been chemically coupled to spectrally addressable polystyrene microspheres or beads. A fluorescent reporter molecule (streptavidin-phycoerythrin) is used to indirectly detect incorporated biotin.

xMAP™ Detection: The fluorescent reporter signals generated for each bead population are read on the Luminex xMAP™ system. Data analysis is performed with Tag-It™ Data Analysis.

Alternative Approaches:

DMET chip Assay: The Drug Metabolizing Enzyme and Transporter (DMET) assay will be used for genotyping identification of all loci of interest (i.e., *CYP2C9; CYP4F2; GGCX; EPHX1; VKORC1*). The process starts with 1) DNA samples dilution to 150 ng/µL in TRIS-EDTA buffer, 2) an initial PCR amplification step, 3) the amplified products are diluted and divided into aliquots, 4) aliquots are combined with 13.4 µL genomic DNA, 5) mixture is incubated with a multiplex PCR mixture containing Targeted Human DMET assay 1.0 probe panel (Affymetrix), 6) Samples will be incubated on the 9700 Thermal Cycler for 4 min at 20°C, 5 min at 95°C, and then overnight at 58°C, 7) Microarrays are washed and scanned with 4-color detection using the Affymetrix GeneChip Scanner 3000 7G 4°C, 8) signal values are normalized and genotypes reported using the Affymetrix GeneChip Operating Software version 1.4 and Affymetrix Targeted Human DMET 1.0 software and 9) Individual samples are assigned a status of “pass” based on routine chip performance metrics.

Sequencing: Coding regions of candidate genes will be bi-directionally sequenced in individual samples by using a next generation sequencer at UPR-MSC facilities (Lab A-640) and called using automated software (Ion-Torrent Personal Genome Sequencer Machine (PGM™) system technology, Semiconductor Sequencing for Life™; Life Technologies Co.). Unknown variants will be manually curated and confirmed by repeat analysis using Sanger method.

PG array: This genotyping assay will be performed using the Illumina BeadArray™ platform and the GoldenGate™ assay. Careful manual analysis will be performed on the alignments underlying the genotype calls using GenCall 6.1.3.24. The Array is composed of 384 SNPs from 222 genes of cardio-metabolic and neuro-endocrine significance. It has been tested on nearly 5,000 patients from various clinical studies. This PG array has been successfully applied in cardiovascular and neuropsychiatric research involving responses to cardiovascular, psychotropic and diabetic drugs as well as to dietary, exercise and acoustical interventions. Public databases (dbSNP, Ensembl) are utilized for validated SNPs with known allele frequencies for mixed or Caucasian populations. SNPs are selected with minor allele frequency consistently between 10% and 30%, while avoiding those with higher allele frequencies, presuming them to be more likely phenotypically neutral. The current analysis is restricted to autosomal genes.

TaqMan SNP assay: The Taqman genotyping assay is designed to detect the presence of small nucleotide polymorphism (SNP) in collected DNA samples. The assay consists in a PCR reaction with specific primers amplifying the polymorphism of interest, followed by two allele specific Taqman probes that will detect the alleles of interest in the reaction (i.e., *CYP2C9*2, *3, *5, *6, *8; EPHX1 rs2234922,* rs1051740, rs2292566, rs4653436, rs2260863; *GGCX* rs12714145; *CYP4F2* rs2108622; *VKORC1*-1639G>A). It uses two fluorescent dyes, VIC and FAM, linked to the 5' end of each allele, and a passive reference dye for normalization purposes. A non fluorescent quencher is placed in the 3' end of the probe allowing for detection of the reporter dye fluorescence with greater sensitivity than with a fluorescence quencher. Cleavage of the probes hybridized to the target sequence by the DNA polymerase (Ampli Taq Gold) separates the quencher from the reporter allowing an increased in the reporter fluorescence signal indicating which alleles are present in the sample.

The DNA sample to be analyzed will be within a concentration between 3ng and 20ng, and it will be mixed together with the 20X Taqman probe, 2X Taqman Universal Master Mix and DNAse free water in order to perform the reaction that will take place in a 96 or 48 well plate and run in the Applied Biosystem Step One Plus Real Time PCR system. The cycling parameters are as followed: 30 seconds at 60°C, 10 minutes at 95°C, 50 cycles of 15 seconds at 92°C and 1 minute at 60°C, and a final step of 30 seconds at 60°C The result is analyzed in the Step One Plus software allowing to interpret the data in the form of a graph containing the analyzed samples organized in three clusters corresponding to the respective genotype, homozygous for allele 1, homozygous for allele 2 and heterozygous.

Statistical Analysis

The primary and secondary hypotheses are listed below accompanied by a description of the statistical procedure used and a justification of sample size for each. The statistical package STATA (ver. 11) or SPSS v11.5 will be used for statistical analyses. Significance will be tested at α=0.05 level.

Hypothesis 1: The allele frequencies of the *CYP2C9, CYP4F2, GGCX, EPHX1* and *VKORC1* genes in the Hispanic population will differ from other populations due to its admixed and heterogeneous ethno-geographic heritage.

Hardy-Weinberg Equilibrium (HWE): Departure from Hardy–Weinberg equilibrium will be estimated under the null hypothesis of the predictable segregation ratio of specific matching genotypes ($p > 0.05$) by use of χ^2 goodness-of-fit test with one degree of freedom. This analysis will be conducted with caution. It is because classical population statistics such as Hardy Weinberg equilibrium may have limited applicability to a population sample of diverse

ethno-geographic ancestry typical of urban populations such as those within metropolitan area of San Juan. Population size is sufficiently large so as to minimize the effect of genetic drift. The population under consideration is idealized because the assumption of random mating. The Hardy-Weinberg principle states the population will have the given genotypic frequencies (called Hardy-Weinberg proportions) after a single generation of random mating within the population. When violations of this provision occur, the population will not have Hardy-Weinberg proportions. The Puerto Rican population is a three-way admixed population that experienced migration (gene flow). No migration assumption is inherent in the Hardy-Weinberg principle and its violation can cause deviations from expectation (i.e., the population will continue to have Hardy-Weinberg proportions each generation, but the allele frequencies will change). However, a violation of assumptions in the Hardy-Weinberg principle does not necessarily mean the population will violate HWE.

Pearson's chi-square test states:
$\chi^2 = \sum (Obs - Exp)^2 / Exp$; where Obs stands for the observed numbers of a given genotype and Exp stands for the expected numbers of a given genotype.

<u>Statistical procedure</u>: After testing for Hardy-Weinberg equilibrium to determine if the alleles are in equilibrium within the population, we will test Hypothesis 1 by comparing the frequency distribution of *CYP2C9**1, and variant *2 and *3 alleles, and the *VKORC1* -1639 GG GA and AA genotypes in our cohort to the frequencies published for various non-Hispanic populations. The mean population frequencies for non-carriers vs. carriers of *CYP2C9*-deficient alleles are 60% and 40%, respectively. For *VKORC1*, published frequencies for the non carriers (GG) and A carriers (GA or AA genotypes) are 52% and 48%, respectively (23).

Sample size estimate for hypothesis 1: A sample of 300 patients is sufficient to test for an absolute difference of 10 percentage points from expected frequency of 59% (i.e., 69% or 49% in outpatients) in *CYP2C9**1/*1 and from an expected frequency of 33% (i.e. 43% or 23% in outpatients) in *VKORC1*GG genotype at $\alpha = 0.05$, $1 - \beta = 0.80$. At $1 - \beta = 0.60$, a 7 percentage point difference can be detected in either gene. Thus, the study is powered to detect major frequency differences and to test the hypothesis that the predominantly Hispanic outpatient population will not be substantially different from published allele surveys in other populations. Corrections have been made by using preliminarily observed frequencies of *CYP2C9* and *VKORC1* polymorphisms in this study population. At present, there is limited or no information published about the frequency distribution of the other markers (*CYP4F2, GGCX,* and *EPHX1*). However, we feel confident that N=300 is enough to test hypothesis 1 even for the less prevalent variants in these genes, as depicted in **Figure 1**.

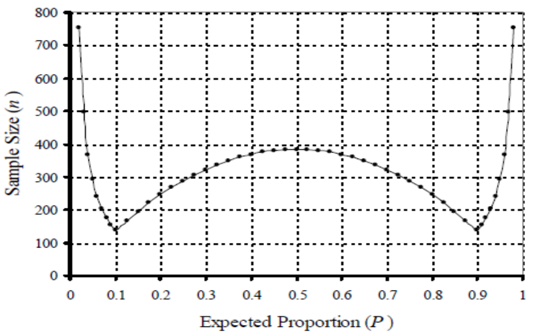

Figure 1 Relationship between Sample Size and Expected Prevalence

Hypothesis 2A: Carriers of *CYP2C9* and/or *VKORC1* polymorphisms will demonstrate lower MD of warfarin compared to those homozygous for the wild type genotype.
Hypothesis 2B: Carriers of *CYP4F2, GGCX,* *EPHX1* variants will demonstrate lower MD of warfarin compared to wild type alleles.
Hypothesis 2C: Carriers of *CYP2C9* and/or *CYP4F2* and/or *GGCX* and/or *EPHX1* and/or *VKORC1* polymorphisms in the Puerto Rican population will demonstrate higher over-anticoagulation risk, higher rate of out-of-range INRs, higher incidence of serious or life-threatening bleeding events and longer time to target therapeutic INR and stable warfarin dosing as compared to wild-type.

Statistical procedures: Analysis of variance using the GLM procedure to accommodate unequal cell sizes will be used to test the effect of genotypes (carrier status) on warfarin maintenance dose (dependent variable). If a significant F ratio is computed, Sidak's post hoc test will be used to determine which groups differ.

Sample size estimate for Hypothesis 2A: In American Caucasians, Linder et al. (24) reported maintenance warfarin dosages of 6.2 ± 2.6 mg/day, 4.4 ± 1.7 mg/day, and 3.64 ± 0.91 mg/day in three groups: *CYP2C9* *1/*1 (wild type, n=34), subjects with one variant allele (either *2 or *3) (n=17), and patients with two variant alleles (n=5), respectively. The published frequencies of patients with the wild type genotype, one variant allele (n=5), and two variant alleles range from 55-69% (21; 24; 27; 31; 32), 33-37% (21; 27), and 4-9% (21; 27; 29), respectively. In the proposed study we will combine patients with one and two variant alleles to allow comparison to those patients with two wild type alleles (*1/*1), unless we discover a large number of Puerto Rican patients with *CYP2C9* *2/*2, *2/*3, or *3/*3 genotypes, in which case we will group them separately and use the GLM ANOVA procedure.

Given the published data (21; 24; 27; 29), our ability to detect a 2 mg/day difference in dose due to genotype, assuming a standard deviation in dose of 2.6 mg/day, requires 11 subjects per group (α=0.01, 1-β=0.80). Preliminarily, we have found that the frequency of the *CYP2C9* wild-type, single and double carriers in the Puerto Rican study population at VACHS are 59%, 31 and 10%, respectively. Accordingly, with 300 subjects expected in the proposed study, we feel certain that the minimum of 11 subjects with ≥1 variant allele will be achieved, and that 11 subjects with two variant alleles are possible (even if some genotype non-calls were to be observed).

Mean warfarin maintenance doses in American Caucasians patients with the *VKORC1* -1639 genotypes GG, GA, and AA were 6.7 ± 3.3 mg, 4.3 ±2.2 mg, and 2.7 ± 1.2 mg, respectively (33-34). Thus the dose was lower by 2.4 mg/day in GA compared to GG, and by 4.0 mg/day in AA compared to GG (33-34). Genotype frequencies were 52%, 36%, and 12% for the *VKORC1*-1639 genotypes GG (wild type), GA, and AA, respectively (33-34). Given these data, we require 8 subjects per genotype to detect a 2 mg/day difference in dose due to genotype, assuming a standard deviation in dose of 2.2 mg/day (α=0.01, 1-β=0.80). Only 4 subjects per group are required for α=0.05 (1-β=0.80). Preliminarily, we have found that the genotype frequencies in the Puerto Rican study population at VACHS were 33%, 52%, and 15% for the *VKORC1*-1639 genotypes GG (wild type), GA, and AA, respectively. Therefore, given the 300 subjects expected in the proposed study, it is reasonable to expect the minimum of 8 subjects per cell even in the less frequent AA genotype.

Sample size estimate for Hypothesis 2B: No preliminary data are currently available about genotype frequencies for polymorphisms within these genes in the Puerto Rican population. However, based on commonly observed prevalence of similar polymorphisms in other populations, we feel confident that N=300 is enough to test hypothesis 2B.

Sample size estimate for Hypothesis 2C: The associations between genotypes and the primary and secondary clinical end-points will be evaluated using survival analysis techniques (Kaplan-Meier time-to-event analysis for anticoagulation-related outcomes and bleeding episodes). To this end, we will follow the same statistical approach used by Higashi et al (31). Two cohorts will be formed based on genotyping information: (1) wild-type and (2) carriers (i.e., having at least one allelic variant). Log-rank test for equality of survivor functions will be performed. The corresponding hazard ratios (HR) and 95% confident intervals (CI) will be calculated for each assessment. Additionally, we plan to use Cox proportional hazards models in order to adjust for potential confounders such as gender, age, comorbid conditions, and drug substrates metabolized by *CYP2C9* (taken by at least 5% of the study population). Notably, this approach is also expected to increase the precision of the developed model. Covariates will be added to the model one at a time to determine potential confounding effects on the variant genotype HR. A covariate will be defined to have an important effect on the HR if the HR changes by more than 5% upon inclusion of the covariate in the model. Duration of warfarin therapy will be measured in days. Patients will be followed up from the index date of first warfarin exposure until either the date of an event observation or the end-of-study date when all data will be subject to administrative right-censoring. Because this study will require a blood sample at enrollment followed by a retrospective medical record review, no patients will be withdrawn or lost to follow-up. To account for changes in the prescribed dose of warfarin, mean daily dose will be programmed as a time varying covariates. Accordingly, the regression model will always use

patients' most recent mean dose to adjust the HR for genotype. In order to assess potential confounding, each covariate will be fitted to the model in order to determine changes to the exposure coefficient. Interaction terms will also be fitted to assess potential effect modification of the genotype exposure by each covariate. Model diagnostics will then be performed and potentially influential cases will be identified. Significant departure from the proportional hazards assumption will be assessed by statistical testing of the Schoenfeld residuals. Stratified analysis on patients' history of above-range INR values will be considered in case of having patients experiencing recurrent above-range INR. Consequently, valid CI will be calculated for recurrent events so that this circumstance does not violate the independence assumption. HR for bleeding events will be calculated during the first 3 months of treatment and during the entire follow-up period. The genotype-associated risk during initiation and over the combined initiation-maintenance phases will be studied. The risk during exclusively the maintenance phase will not be assessed because this would involve recoding time zero at a later point, which could introduce bias if carriers have more first bleeding events during initiation and are thus censored, and would not allow to define the time until first bleeding event following warfarin administration. An unadjusted incidence rate ratio will also be calculated for bleeding events by taking the ratio of the unadjusted bleeding rates in the wild-type and variant genotype groups.

Hypothesis 3: *CYP2C9, CYP4F2, GGCX, NQO1, EPHX1* and *VKORC1* genetic variants will significantly predict the optimal therapeutic dose of warfarin, i.e., that needed to produce an INR in the range of 2.0 to 3.0 in the VACHS population.

Statistical procedures: If warfarin dose is not normally distributed, we will apply a log transformation of the variable. We will construct an inter-correlation matrix to identify genetic, demographic, and other clinical variables that correlate with warfarin dose. If warfarin dosage differs across genotype groups as expected (Hypothesis 2A and B), then Hypothesis 3 is testable *initially* by applying multiple regression using maintenance warfarin dose as the dependent variable and the numerical designations of "1" for the wild-type genotype; "2" for the single-carrier genotypes; and "3" for double-carrier genotypes of each of the candidate genes. Forward addition and backward elimination regression procedures (stepwise) to build regression models will be applied. The following criteria will be used for inclusion of new variables in the model: probability of F to enter, p=0.05-0.10, and the probability of F to remove, p=0.15-0.20.

Sample size estimate for Hypothesis 3: A robust multiple regression analysis requires > 15 cases per independent variable. The expected size of our cohort (N=300) suggest we may safety investigate 15 or even more independent variables. If significant effects for genotypes are observed (see Hypothesis 2A & B), we will have a context to evaluate their independent contributions. In English (21) and American Caucasians (20), four significant predictors of

warfarin dosage were found: *CYP2C9* genotype, *VKORC1* genotype, age, and a measure of body size (height, weight). We will begin but not limit our investigation to these four variables. In addition, sex/gender, concomitant medications, Vitamin K intake, initials INRs and admixture indexes, as well as their interactions, will also be investigated as independent predictors.

Hypothesis 4A: A low and inconsistent intake of dietary vitamin K will contributes to intra-individual variability in anticoagulation response, with those with unstable control of anticoagulation having a lower or more variable intake than those with stable control.
Hypothesis 4B: Unstable anticoagulation control due to low intake of dietary vitamin K is more likely to happen and more severe in those carriers of the *VKORC1*-1639G>A variant.

Each patient eligible to participate in this study after the approval's date of protocol's amendment #3 (March, 2010), will be provided with a vitamin K-oriented intake food questionnaire, with a previously developed food scale and weighing factors, and the same set of detailed instructions on how to complete the questionnaire in order to maximize the consistency and accuracy of dietary recording. A number of previously validated photographs of commonly eaten foods will be included with the questionnaire and used to estimate portion sizes. At the end of each week the patients will be seen at the clinic, where dietary recall approaches will be used when incomplete descriptions of food are reported. If a patient is unable to give details about either the weight or portion size then the weight of food eaten will be approximated using average portion sizes as early described by Sconce *et al* (47-48).

Statistical procedures: STATA program (version 11) will be used to carry out statistical analysis of the data. Logarithm-transformed values will be used for comparing stable with unstable patients in the event of any skewed distribution of data is observed. Simple correlation of changes in vitamin K intake between week 1 and 2 of the study and changes in INR in unstable patients will be performed. Two-sample t-tests will be performed to compare the mean intake of vitamin K (scores) of stable and unstable patients. Standard deviation (±SD) of vitamin K intake scores and variation about the mean will be used as a measure of variability of intake. Stratification by genotyping (*VKORC1*) and linear regression analysis (all raw data and carriers/non-carriers) using vitamin K scores versus time to stable INR will be carried out to investigate the role of *VKORC1*-1639G>A polymorphism in the expected association (i. e., as a confounder or effect modifier). Hazard ratios (HR) will be estimated. Since about 40% of Puerto Rican patients are at least single carrier of *VKORC1* polymorphisms (49), we expect to have no less than 10 patients in each group showing this risk factor. To this end, a categorical variable will be created (0, wild-type; 1, carriers of *VKORC1* variant). Pearson's correlation test will also be performed to identify whether there is a relationship between changes in vitamin K intake scores and changes in INR (time to

stable INR). Results will be presented as mean ± SD unless otherwise is required. A p-value of <0.05 will be taken as statistically significant.

Sample size estimate for hypotheses 4: A sample size of 35 patients is estimated to have 80% power to detect a hazard ratio (HR) of 0.3 at a significant level α=0.05 (2-tailed). The required sample size is calculated based on Schoenfeld's formula for the proportional hazards regression models, using the Vanderbilt University's PS software (ver. 3.0.12), which is available online at http://biostat.mc.vanderbilt.edu/PowerSampleSize. We are planning a study with an expected ratio of 0.6 wild-types per patient carrying at least one allelic variant in VKORC1 gene locus, given the observed 35-40% frequency distribution of carriers for VKORC1 variants in previous studies with Puerto Ricans. In a previous study, the median time to achieve daily warfarin dose stabilization on the wild-types patients was approximately 0.25 years. If the true hazard ratio of wild-type subjects relative to carriers is 0.3, we will need to study 25 carriers and 15 wild-type patients (N=35) to be able to reject the null hypothesis that the variant and wild-types are equal with a probability of 80% (power). The Type I error probability associated with the test of this null hypothesis is α=0.05.

Cost-effectiveness assessment

Application of warfarin pharmacogenetics is controversial and high dependent on its cost-effectiveness. The current high cost of DNA testing to guide warfarin dose determination opens the question whether pharmacogenetics is cost-effective when implement warfarin treatment. The cost of pharmacogenetic screening for *CYP2C9* and *VKORC1* is approximately US $350 -400 (41). Yet, the cost-effectiveness of using pharmacogenetic information in warfarin treatment is not well-understood (41-46). The cost per INR test in Puerto Rico (CPT 85610) is approximately US $10 per test. Usually, a patient taking warfarin is tested at least nine times (around US $90) per year. To assess the real cost of this testing it is important to consider the cost incurred in adjusting the warfarin dose and the cost to treat adverse drugs reactions. Patients carrying multiples combinatorial polymorphisms on CYP2C9 and *VKORC1* genes take as average between 15-20 days to find the correct dose with a stable INR and need to be tested more frequently than usual to archive an adequate dose. Although some studies have concluded that warfarin pharmacogenomics is unlikely to be cost-effective for general patients at present, other authors have found different influential factors to improve the cost-effectiveness of warfarin genotyping, including low genotyping cost, faster turnaround time, high effectiveness in improving anticoagulation control/event rate, and applying warfarin pharmacogenomics to patients with high bleeding risk or poor anticoagulation control.

The purpose of this research is to compare the cost-effectiveness of INR monitoring in patients with high risk of adverse drug reactions and compare it to the hypothetical cost-

effectiveness of implementing pharmacogenetic determination. Our hypothesis is that, in the VACHS-affiliated anticoagulation clinic, the cost effectiveness of implementing genetic testing in high risk patients is better than the current cost-effectiveness of INR monitoring. Therefore, the costs of hospitalization and treatment for adverse reactions will be reduced by implementing *CYP2C9* and *VKORC1* genotyping in patients who failed to achieve stable warfarin dose during the initial phase of therapy.

This is a cost-effectiveness retrospective study in Puerto Rican patient on stable warfarin therapy in the VACHS-affiliated anticoagulant clinic from the perspective of the healthcare provider (i.e., VACHS), to project the cost, major bleeding/thromboembolic event rate in first year of warfarin therapy. We plan to analyze the 300 patients to be enrolled in this protocol, as described in section B “Sample”, on page 16, in order to perform a secondary analysis from collected data of the research protocol.

Data extraction: Relevant data for this analysis will be extracted from both the patients' medical records using the computerized patient record system (CPRS) and the already existing master database created by investigators of this ongoing protocol. We will extract only the medical information relevant to calculate the cost-effectiveness of INR monitoring and genetic testing. Individual medical records and database will be revised for clinical information regarding cost-effectiveness considering a time period of one year long, starting at the date of the first reported warfarin dose for each patient. The information to be extracted will be the following:

1. The date (mm/dd/yy) patient received the first dose of warfarin
2. The number of INR testing required to reach a stable warfarin dose, as previously defined in section C “Enrollment Criteria” on page 16
3. Adverse drug reactions (ADRs) reported during the initial phase of warfarin therapy (e.g., bleeding, strokes, etc.)
4. Number of Hospitalization
5. Concomitant disease
6. Medications received during the initial anticoagulation therapy (drug interactions)
7. How were the ADRs treated?
    a. Adjust warfarin dose
    b. Administrating Vitamin K
    c. Need blood transfusion
    d. Interaction with other medication
8. The genotype information for *VKORC1* and *CYP2C9*

The estimated costs of hospitalizations, treatment of warfarin-induced ADRs, dose adjustments, INR measurements, transportation, genotypes, concomitant medications and other critical variables of economic interest will be obtained by consulting the VACHS

administrative office, Genomas-Laboratory of Personalized Health or from the available literature.

Analysis: The decision analytical model that we propose considers the actual monitoring of oral anticoagulant (INR levels) and the second branch is DNA-guide warfarin dosing. The DNA-guide warfarin dosing has three branches: (1) single carrier (2) multiple carrier (3) wild-type. At the end of the model are showed three outcomes, the first is the probability to develop stroke, the second is the incidence related to bleedings and other ADRs and the last one is the probability to not develop any complication. See **Figure 2** below.

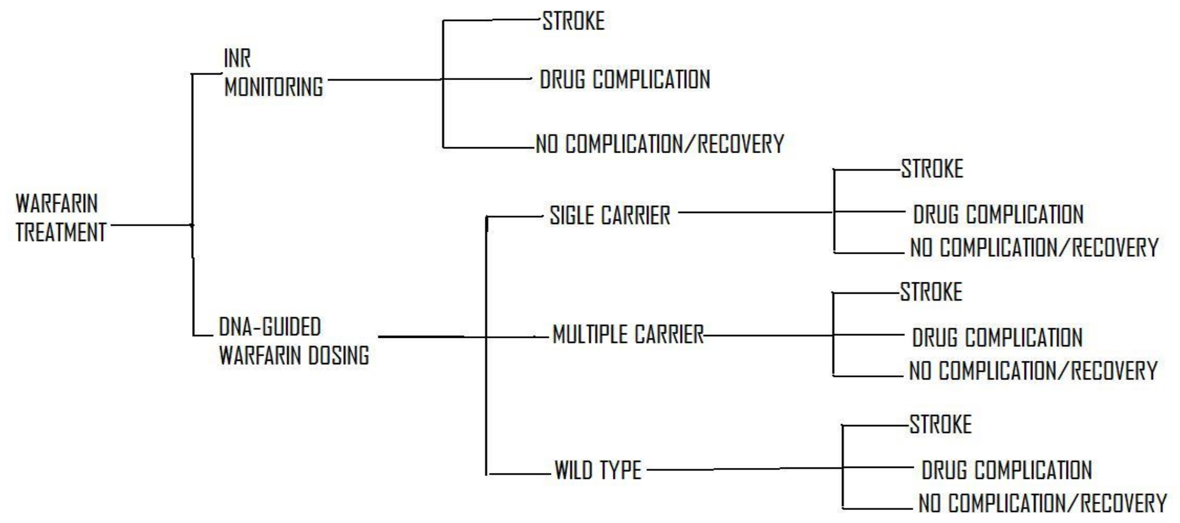

Decision Tree Analytical Model

Using the decision analytical model we will able to compute the expected (average) medical care costs per patient under INR monitoring and DNA-guided warfarin dosing for 12 month period after the initial treatment. Analyses will include direct medical costs (e.g. costs of genotyping, warfarin therapy, anticoagulation care, and treatment of major bleeding and thromboembolic events, all US cost data). Other outcomes measurements such as cost per event averted and cost per life saved will also be considered. To determine if exist any relationship between the incidence of developing an adverse drug complication (bleeding), genetic status of the patient, the record of stroke and number of hospitalization, concomitant disease and the variation of INR levels, we will perform a logistic regression analysis. The costs incurred to treat the oral anticoagulant therapy will be tabulate in a spreadsheet and the formula used to calculate the cost-benefit ratio is:

Benefit-Cost Ratio = (Cost averted by intervention)/ (Cost of intervention)

In this formula the benefits are medical care expenditures saved by DNA-guided warfarin dosing compared to INR monitoring, if any, and, costs are those costs related to the DNA-guided warfarin dosing intervention.

Preliminary Results

Recent assessment of the CYP2C9 and VKORC1 alleles in Puerto Ricans: Preliminary results were obtained after genotyping 100 DNA samples from the Puerto Rican Newborn Screening program, using xMAP Luminex100 technology HILOmet DNA typing system (test code 70004) at the LPH-Genomas. The polymorphism frequencies were 6.52%, 5.43% and 28.8% for the functionally deficient *CYP2C9**2, *CYP2C9**3 and *VKORC1* -1639 G>A polymorphisms, respectively. Notably, one sample from this study population carried the uncommon allele CYP2C9*6, which is associated with decreased enzyme activity. CYP2C9*6 (results from 818delA) was first described in a female African American and is regularly related to black African descendants. Combining prevalence of combinatorial genotypes, 16% were carriers of both *CYP2C9* and *VKORC1* polymorphisms, 9% were *CYP2C9* polymorphism carriers only, 35% were *VKORC1* carriers only, and the remaining 40% were non-carriers for either gene. Based on published warfarin dosing algorithm, single, double and triple carriers of functionally deficient polymorphisms predict reductions of 1.0 to 1.6, 2.0 to 2.9, and 2.9 to 3.7 mg/day, respectively, in warfarin dose (20-21). Overall, 60% of the study population (6 in 10 individuals) carried at least a single polymorphism predicting deficient warfarin metabolism or responsiveness and 13% were double carriers with polymorphisms in both genes studied. VKORC1 promoter polymorphism has been associated with a 28% [95% CI: 25% to 30%] decrease in the warfarin dose per allele (www.WarfarinDosing.org). All warfarin-resistant patients carried the G allele at –1639 while none of the warfarin-sensitive patients did (19; 35). This SNP correlated with gene expression of vitamin K epoxide reductase activity suggesting that carriers of the A allele require lower warfarin doses because they have lower endogenous epoxide activity. This combinatorial genotyping of *CYP2C9* and *VKORC1* can allow for individualized dosing of warfarin amongst patients with gene polymorphisms potentially reducing the risk of stroke or bleeding.

In addition, our preliminary findings in the study population at VACHS revealed that 69% of patients were carriers of at least one polymorphism in either *CYP2C9* or *VKORC1* gene. Double, triple and quadruple carriers accounted for 22%, 5% and 1%, respectively. We found two carriers of the CYP2C9*5 allele, which is a rare loss-of-function variant that is frequently found in African descendants. No significant departure from HWE was found. Among patients with a given *CYP2C9* genotype, warfarin dose requirements declined from GG to GA and AA haplotypes; whereas, within each *VKORC1*-1639G>A genotype, the warfarin

dose decreased as the number of *CYP2C9* variants increased. The presence of these loss-of-function alleles was associated with more out-of-range INR measurements (OR=1.38) but not with significant INR>4 during the initiation phase. Based on a published algorithm, carriers of functionally deficient polymorphisms predict reductions of up to 4.9 mg/day in warfarin dose.

Currently, we have performed a regression analysis to develop a pharmacogenetic driven warfarin dosing algorithm in 163 Puerto Ricans patients. As observed, our customized model in Puerto Ricans is showing a better predictability than previously published algorithms in other populations (R^2=0.678 and a mean absolute difference of only 0.79 mg/day). However, common variants in candidate genes explained <12% of dose variability. Given the fact that genotyping account for up to 30% of dose variability in available algorithms, we believe that further analysis is required to identify and incorporate such “missing heritability” in our model.

Recent policies at Federal Drug Administration (FDA) incorporate DNA typing information for save and effective drug prescribing (37). The FDA Clinical Pharmacology Advisory Sub-committee in November 2005 recommended revising the Warfarin label to include DNA typing of both CYP2C9 and VKORC1 based on evidence that lower doses are needed for patients with certain gene variants. A label revision with both genes is expected in 2007. Healthcare will be revolutionized by DNA-guided medicine in clinical practice. The practice of personalized medicine will be increasingly dependent on defining innate drug metabolizing capacity by DNA typing and managing therapy on an individualized basis (38).

References

1. Ansell J, Hirsh J, Poller L, Bussey H, Jacobson A and Hylek E. The pharmacology and management of the vitamin K antagonists: the Seventh ACCP Conference on Antithrombotic and Thrombolyc Therapy. Chest 126: 204S-233S, 2004.
2. McWilliam, A., Lutter, R., Nardinellli, C. Health care savings from personalized medicine using genetic testing: the case of warfarin. Working Paper 06-23. 2006. AE1-Brookings Joint Center for Regulatory Studies. 2006.
3. Linkins LA, Choi PT and Douketis JD. Clinical impact of bleeding in patients taking oral anticoagulant therapy for venous thromboembolism: a meta-analysis. Ann Intern Med 139: 893-900, 2003
4. Ansell JE, Shepro DS. Anticoagulation-Advisor. Hudson Physician Communications, Inc. July, 2001.
5. Roberts GW, Helboe T, Nielsen CBM, Gallus AS, Jensen I, Cosh DG, Eaton VS. Assessment of an Age-Adjusted Warfarin Initiation Protocol. Ann Pharmacother 2003; 37: 799-803.
6. Roberts GW, Druskeit T, Jorgensen LE, Wing LM, Gallus AS, Miller C, Cosh D, Eaton VS. Comparison of an age-adjusted warfarin loading protocol with empirical dosing and Fennerty's protocol. Aust NZ J Med 1999; 29: 731-6.
7. Harrison L, Johnston M, Massicotte MP, Crowther M, Moffat K, Hirsh J. Comparison of 5 mg and 10 mg loading doses in initiation of warfarin therapy. Ann Intern Med 1997; 126: 133-6.
8. Crowther MA, Harrison L, Hirsh J. Warfarin: Less may be better. Ann Intern Med 1997; 127: 333.
9. Crowther MA, Ginsberg JB, Kearon MB, et al. A randomized trial comparing 5 mg and 10 mg warfarin loading doses Ann Intern Med 1999; 159: 46-48
10. von Eschenbach AC. CMPI Conference: Panel on the Politics of Drug Evaluation, Washington, DC, Feb 21, 2007, http://www.visualwebcaster.com/event.asp?id=37936. Accessed on March, 2008
11. Lesko LJ. Presentation at the ASHP Midyear Clinical Meeting Educational Session, Las Vegas, NEV, Dec 2005. http://www.ashp.org/s_ashp/article_news.asp?CID=167&DID=2024&id=13520. Accessed on April, 2007.
12. Takahashi H, Wilkinson GR, Nutescu EA, et al. Different contributions of polymorphisms in VKORC1 and CYP2C9 to intra- and inter-population differences in maintenance doses of warfarin in Japanese, Caucasians and African Americans. Pharmacogenet Genomics. 2006; 16:101-110.
13. Sanderson S, Emery J, Higgins J. CYP2C9 gene variants, drug dose, and bleeding risk in warfarin-treated patients: A HuGEnet™ systemic review and meta-analysis. Genet Med. 2005; 7:97-104.

14. Wandelius M, Chen LY, Downes K, Ghori J, Hunt S, Ericksson N, Wallerman O, Melhus H, Wadelius C, Bentley D and Deloukas P. Common VKOR1 and GGCX polymorphisms associated with warfarin dose. Pharmacogenomics J 5: 262-270, 2005.
15. Veenstra DL, You JHS, Rieder MJ, et al. Association of Vitamin K epoxide reductase complex 1 (VKORC1) variants with warfarin dose in a Hong Kong Chinese patient population. Pharmacogenet Genomics. 2005; 15-687-691.
16. Wadelius M and Pirmohamed M. Pharmacogenetics of warfarin: current status and future challenges. Pharmacogenomics J 2006.
17. Hampton T. Researchers draft guidelines for clinical use of pharmacogenomics. JAMA 296: 1453-1454, 2006.
18. Gage BF, Eby C, Milligan PE, Banet GA, Duncan JL, McLeod H.. Use of pharmacogenetics and clinical factors to predict the maintenance dose of warfarin. Thromb Haemost 2004; 91:87–94.
19. Gage BF. Pharmacogenetics-Based Coumarin Therapy. Hematology 2006; 1: 467-473.
20. Zhu Y, Shennan M, Reynolds KK, Johnson NA, Herrnberger MR, Valdes R Jr., Linder MW. Estimation of Warfarin Maintenance Dose Based on VKORC1 (-1639 G>A) and CYP2C9 Genotypes. Clinical Chemistry 2007; 53: 1199-1205.
21. Sconce EA, Khan TI, Wynne HA, Avery P, Monkhouse L, King BP, Wood P, Keteven P, Daly AK and Kamali F. The impact of CYP2C9 and VKORC1 genetic polymorphism and patient characteristics upon warfarin dose requirements: proposal for a new dosing regimen. Blood 106: 2329-2333, 2005.
22. Li T, Chang CY, Jin DY, Lin PJ, Khvorova A and Staford DW. Identification of the gene for vitamin K epoxide reductase. Nature 427: 541-544, 2004.
23. Couris R, Tataronis G, McCloskey W, Oertel L, Dallal G, Dwyer J and Blumberg JB. Dietary vitamin K variability affects International Normalized Ratio (INR) coagulation indices. Int J Vitam Nutr Res 76: 65-74, 2006.
24. Linder MW, Looney S, Adams JE, III, Johnson N, Antonono-Green D, Lacefild N, Bukaveckas BL, and Valdes R, Jr. Warfarin dose adjustments based on CYP2C9 genetic polymorphisms. J Throb Thrombolysis 14:227-232, 2002.
25. Hylek EM. Oral anticoagulants. Pharmacologic issues for use in the elderly. Clin Geriatr Med 17: 1-13, 2001.
26. Absher RK, Moore ME and Parker MH. Patient-specific actors predictive of warfarin dose requirements. Ann Pharmacother 36: 1512-1517, 2002.
27. Scordo Mg, Pengo V, Spina E, Dahl ML, Gusella M and Padrini R. Influence of CYP2C9 and CYP2C19 genetic polymorphism on warfarin maintenance dose and metabolic clearance. Clin Pharmacol Ther 72: 702-710, 2002.
28. Mushiroda T, Ohnishi Y, Saito S, Takahashi A, Kikuchi Y, Saito S, Shimomura H, Wanibuchi Y, Suzuki T, Kamatani N and Nakamura Y. Association of VKORC1 and

CYP2C9 polymorphisms with warfarin dose requirements in Japanese patients. J Hum Genet 51: 249-253, 2006.

29. Wang SL, Huang J, Lai MD and Tsai JJ. Detection of CYP2C9 polymorphism based on the polymerase chain reaction in Chinese. Pharmacogenetics 5: 37-42, 1995
30. Gordon J, Ferante S, Weiss J, et al. Pharmacogenetic P450 screening using the Tag-It ^{TM} Universal Bead-Based Array Platform. Edited by Valdes R, Jr.,Linder MW and Wong S. AACC Press, 2006.
31. Higashi MK, Veenstra DL, Kondo LM, Wittkowsky AK, Srinouanprachanh SL, Farin FM, Rettie AE. Association between CYP2C9 genetic variants and anticoagulation-related outcomes during warfarin therapy. JAMA 287: 1690-1698, 2002.
32. Wadelius M, Sorlin K, Wallerman O, Karlsson J, Yue QY, Magnusson PK, Wadelius C, Melhus H. Warfarin sensitivity related to CYP2C9, CYP3A5, ABCB1 (MDR1) and other factors. Pharmacogenomics J 4: 40-48, 2004.
33. D'Andrea G, D'Ambrosio RL, Di Perna P, et al. A polymorphism in the VKORC1 gene is associated with an inter-individual variability in the dose-anticoagulant effect of warfarin. Blood 2005; 105: 645-649.
34. Marsh S, King CR, Porche-Sorbet RM, Scott-Horton TJ, Eby CS. Population variation in VKORC1 haplotype structure. J Thromb Haemost. 2006; 4:473–474.
35. Crawford DC, Ritchie MD, Rieder MJ. Identifying the genotype behind the phenotype: a role model found in VKORC1 and its association with warfarin dosing. Pharmacogenomics. 2007, 8(5): 487-496.
36. Rieder MJ, Reiner AP, Gage BF, Nickerson DA, Eby CS, McLeod HL, Blough DK, Thummel KE, Veenstra DL, Rettie AE. Effect of VKORC1 haplotypes on transcriptional regulation and warfarin dose. N Engl J Med 2005; 352:2285-93.
37. Womack, C. As Part of Retrofitting, FDA Panel Votes to Relabel Warfarin for PGx; Is Dx Far Behind? Pharmacogenomics Reporter. 2005. 12-1-0005.
38. Ruaño G. Quo Vadis Personalized medicine? Personalized medicine 1: 1, 2004.
39. Rosenberg Y. Rationale and outline of an NHLB/NIH Initiative for a large, multicenter randomized trial of genotype-guided dosage of warfarin therapy. Slide presentation NHLB/NIH workshop: 2006.
40. Xie HG, Prasad HC, Kim RB and Stein CM. CYP2C9 allelic variants: ethnic distribution and functional significance. Adv Drug Deliv Rev 54: 1257-1270, 2002.
41. Eckman MH, Rosand J, Greenberg SM, Gage BF. Cost-effectiveness of using pharmacogenetic information in warfarin dosing for patients with non-valvular atrial fibrillation. Ann. Intern. Med. 2009; 150(2): 73-83.
42. Bushnell CD, Datta SK, Goldstein LB. Cost implications of specialized coagulation testing for acute ischemic stroke. Journal of Stroke and Cerebrovascular Diseases: The Official Journal of National Stroke Association. 2001; 10(6): 279-283.
43. Jowett S, Bryan S, Mahé I, et al. A multinational investigation of time and traveling costs in attending anticoagulation clinics. Value in Health: The Journal of the

International Society for Pharmacoeconomics and Outcomes Research. 2008; 11(2): 207-212.

44. Leey JA, McCabe S, Koch JA, Miles TP. Cost-effectiveness of genotype-guided warfarin therapy for anticoagulation in elderly patients with atrial fibrillation. Am. J. Geriatr. Pharmacother. 2009; 7(4): 197-203.
45. Sorensen SV, Dewilde S, Singer DE, Goldhaber SZ, Monz BU, Plumb JM. Cost-effectiveness of warfarin: Trial versus "real-world" stroke prevention in atrial fibrillation. Am. Heart J. 2009; 157(6): 1064-1073.
46. Sullivan PW, Arant TW, Ellis SL, Ulrich H. The cost effectiveness of anticoagulation management services for patients with atrial fibrillation and at high risk of stroke in the US. Pharmacoeconomics. 2006; 24(10): 1021-1033.
47. Sconce E, Khan T, Mason J, Noble F, Wynne H, Kamali F. Patients with unstable control have poorer dietary intake of vitamin K compared to patients with stable control of anticoagulation. Thromb Haemost 2005; 93: 872-5.
48. Sconce E, Avery P, Wynne H, Kamali F. Vitamin K supplementation can improve stability of anticoagulation for patients with unexplained variability in response to warfarin. Blood 2007; 109(6):2419-23
49. Duconge J, Cadilla CL, Windemuth A, Kocherla M, Gorowski K, Seip RL, Bogaard K, Renta JY, Piovanetti P, D'Agostino D, Santiago-Borrero PJ, Ruaño G, 2009 Ethnicity and Disease, 2009;19(4):390–395
50. Adjusted-dose warfarin versus low-intensity, fixed-dose warfarin plus aspirin for high-risk patients with atrial fibrillation: stroke prevention in atrial fibrillation III Randomized clinical trial. Lancet 1996; 348:633-8
51. Gage BF, Cardinalli AB, Owens DK. The effect of stroke and stroke prophylaxis with aspirin or warfarin on quality of life. Arch Intern Med 1996;156:1829-36
