# Supplementary material for: A Novel Admixture-Based Pharmacogenetic Approach to Refine Warfarin Dosing in Caribbean Hispanics
Source: PLoS One. 2016 Jan 8;11(1):e0145480. doi: 10.1371/journal.pone.0145480 (PMC4706412; doi:10.1371/journal.pone.0145480)
Supplement: S2 Text — (PDF) [file pone.0145480.s002.pdf]

Subject Name: ______________________________ **Date:** __________________

Title of Study: ___Pharmacogenetic-driven Warfarin Dosing Algorithm in Puerto Ricans_________

Principal Investigator: Giselle Rivera,
Juan F. Feliu,
Jorge Duconge

VA Caribbean Healthcare System
10 Casia Street
San Juan, PR 00921-3201

Sponsor: NIH

1. This paragraph indicates that I have knowledge about my condition:
You qualify to participate in this investigation at the VA Caribbean Healthcare System (VACHS) because you are a patient treated with warfarin, a blood thinner or anticoagulant, and receive follow–up care in the Anticoagulation Clinic at this institution. Patients that use warfarin, which is an agent to help prevent and treat coagulation problems, meet the criteria to participate in this study. You need close monitoring when taking warfarin because, taking too much drug may increase the chances of bleeding and not taking enough drug may increase the chances of a blood clot or a stroke. Some people are sensitive to warfarin which means that they have a higher response than expected. This sensitivity increases the risks of bleeding problems.

This protocol will include Puerto Rican patients in anticoagulant therapy at the Anticoagulation Clinic. Your participation in this study is entirely voluntary. You should read the information below, and ask questions about anything you do not understand before deciding whether to participate.

2. Purpose of the study and how long it will last: Warfarin is an anticoagulant or "blood thinner" commonly taken by patients like you in order to prevent blood clots. Even though most drugs have a standard effective dose, warfarin doses may vary significantly between patients. The effectiveness of warfarin is monitored by a laboratory test known as the INR. This test is used to adjust the doses of warfarin and decrease the risks of bleeding. It may take a couple of weeks or months to achieve appropriate anticoagulation levels on each patient.

Sensitivity to warfarin may run in families. To determine this, scientists study the genes of the individuals. You inherit your genes from your parents and these genes play a role in defining many of the personal traits of each individual, such as height and color of eyes. The genes are found in cells within your blood and specifically are contained in the DNA (deoxyribonucleic acid) portion of you blood cells. Scientists can use your DNA to identify traits inherited for heart diseases and coagulation factors, bleeding and sensitivity to anticoagulant agents. Genes can also explain who can be sensitive to warfarin therapy and what treatment best functions for each individual.

This study will look at genetic variations among patients taking warfarin by means of a blood test. The purpose of this assessment is to determine if your personal DNA is a good predictor of your correct warfarin dose. If this is found possible, then an appropriate warfarin dose will be calculated in lesser time.

Approximately 300 patients on warfarin are expected to participate in this study. The participation of each patient, including you, will last about an hour. The study will last about three to four years.

SUBJECT'S IDENTIFICATION (I.D. plate or give name - last, first, middle)

NOMBRE: ________________________________
SS#: ___/___/_____

(check one box)

☐ Initial Consent Subject #: _________

☐ Re-consented

VACHS IRB APPROVED
10/21/2013

Subject Name: ________________________________ **Date:** ___________________
Title of Study: ___Pharmacogenetic-driven Warfarin Dosing Algorithm in Puerto Ricans_________
Principal Investigator: Giselle Rivera, Juan F. Feliu, Jorge Duconge
VA Caribbean Healthcare System
10 Casia Street
San Juan, PR 00921-3201
Sponsor: NIH

3. Description of the study including procedures to be used:

During your visit your health care provider in the Anticoagulation Clinic will performed the first approach on participation in the study. If you wish to participate, you will meet with one of our researchers who will discuss the aspects of our study. Your take will be discussed and given written consent in which agree to participate in the study. Researchers of this study take out an appointment to meet in the lab and take a sample of 5 ml of blood.

- The researchers will ask you to complete a questionnaire and ask you questions about your ethnic background and ancestry during one of your Anticoagulation Clinic visits.
- During your visit to the clinic, researchers from this study may ask you about the quantities of vitamin K in your diet. Information about your medical records may be collected including: age, gender, height, weight, history about warfarin therapy, and the current list of medications you are taking, health conditions and evidence of prior complications due to bleeding episodes or other diagnoses. In case this information does not appear on your medical record, it may be requested. At the end of the study all information will be stored in a file in the Research Department.
- A small blood sample will be taken from you, (approximately a teaspoon), for DNA analysis during the same scheduled laboratory appointment for routine sampling and monitoring of your anticoagulation INR determination. This means no additional punctures are needed. The investigators will code your blood sample with a unique study number to identify the blood sample and protect your personal information.

The coded blood sample will be sent to a certified and specialized laboratory for your DNA analysis. The researchers at this laboratory will not have access to any information that might identify you. There are no plans to change your treatment based on the results of this analysis. Investigators in this study do not plan to inform you about the results of your DNA analysis, even though you have the right to request this information. Please inform the investigators if you wish to receive information regarding the test results. The VACHS (Dra. Giselle Rivera and Dr. Juan Feliu) will keep secured any information that might reveal your identity. This information will be kept in a separate file from the study data. The original list and any study information will be stored in a secure place in the Veteran's Hospital, Pharmacy Service Office 119.

All the collected blood samples from participants like you will be destroyed by the clinical laboratory once the study has concluded. Blood samples will not be used with any other purpose than the described above. The original code list will be destroyed as soon as all data is collected for the study and the Hospital authorizes its destruction. Once this is completed, there will be no way of knowing which was your sample.

This study is experimental; therefore the blood tests and data collection will be used only for investigation purposes.

VACHS IRB APPROVED
10/21/2013

Subject Name: ______________________________ **Date:** __________________

Title of Study: ___Pharmacogenetic-driven Warfarin Dosing Algorithm in Puerto Ricans________

Principal Investigator: Giselle Rivera, Juan F. Feliu, Jorge Duconge

VA Caribbean Healthcare System
10 Casia Street
San Juan, PR 00921-3201

Sponsor: NIH

4. Description of any procedures that may result in discomfort or inconvenience:

You may experience mild physical discomforts as a result of participating in this research study. Even though this study does not require making additional punctures other than those planned for your follow-up routine testing, the blood extraction may cause you some discomfort due to the puncture of the vein.
The blood sample collection might cause slight stress, anxiety and discomfort, particularly during the needle stick. There is a small chance that you might experience some bruising or tenderness at the spot where the blood is taken. Pressing hard on the spot for 1 or 2 minutes after the needle is removed will help to prevent a bruise.

Occasionally, people feel lightheaded or even faint when their blood is drawn. If you feel dizzy, tell the person collecting the blood sample and he/she will help you lay down until the discomfort has disappeared.

A laboratory test always causes certain emotional tension before and after.

5. Expected risks of study:
The risks of sharing your DNA results with your employer and/or any insurance company representative, like your health insurance company, are minimal since this personal information is confidential and therefore protected by the principal investigators of the study and will not be shared with other persons outside the study. Only the investigators of this study will have access to your personal information, including the existing in your medical records and that obtained by the procedure performed in the study, which will be used only for the purposes of this study. It is possible that an authorized member of the VACHS and/or a member of the safety monitoring committee that protects human rights in this study can also request access to your information derived form the study, but only for control or monitoring purposes of our work.

Federal laws and policies provide you with protection from discrimination by health insurance companies, group health plans, and most employers based on your genetic information. A new federal law, the Genetic Information Nondiscrimination Act (GINA) generally will protect you in the following ways:

- Health insurance companies and group health plans may not request your genetic information obtained from this research.
- Health insurance companies and group health plans may not use your genetic information obtained from this research when making decisions regarding your eligibility or premiums.
- Employers with 15 or more employees may not use your genetic information obtained from this research when making a decision to hire, promote, or fire you or when setting the terms of your employment.

Be aware that this new Federal law does not protect you against genetic discrimination by companies that sell life insurance, disability insurance, or long-term care insurance.

VACHS IRB APPROVED

10/21/2013

Subject Name: ________________________________ **Date:** ___________________

Title of Study: ___Pharmacogenetic-driven Warfarin Dosing Algorithm in Puerto Ricans_________

Principal Investigator: Giselle Rivera, Juan F. Feliu, Jorge Duconge

VA Caribbean Healthcare System
10 Casia Street
San Juan, PR 00921-3201

Sponsor: NIH

6. Expected benefits of study: There may be no direct benefit to you from participating in this study. But, it is possible that the information learned during this study may help us better understand how to properly calculate the warfarin dose. This will ultimately help future patients that need warfarin therapy to receive their optimal dose and achieve proper blood anticoagulation levels and clinical outcomes.

7. Other treatment available: This is not a treatment study. You may choose not to participate in this study without any penalty to you. However, if you choose not to participate, you will receive your usual follow-up for treatment of your condition with warfarin.

8. Use of research results: The information derived from this study may be incorporated into research databases so that they can be later used to study better measures of safety and effectiveness in warfarin therapy, design other dosing algorithms for patients, gain a better understanding of disease management, or improve the efficacy of future clinical trials. The results can be used for future publications. To ensure the scientific integrity of the study, you agree that you may not be able to review some of your records related to the study until after the study has been completed.

9. **Compensation**

You will not be paid for your participation or donating your blood sample, nor for physical injuries or salary lossess during participation in this study. You will not have to pay additional costs for participating in this research.

In the event that you sustain a physical injury as a direct result of your participation in this study, all necessary and appropriate care will be provided without charge at the VACHS. You will sign VAF 10-1086.

You will not be required to pay for treatment received as part of your participation in a VACHS research program. However, veterans in some categories may be subject to copayments if indicated by a means test, in accordance with Federal Regulations.

10. Confidentiality

You will give a small blood sample for DNA analysis (approximately one teaspoon). The investigators will code your blood sample with a unique study number in order to protect your identity. The sample will be analyzed at the Molecular Genetics Laboratory at the University of Puerto Rico Medical Sciences Campus.

Your identity will not be disclosed to the public. However, your medical records may need to be reviewed by the members of the Research and Development Committee and the Human Studies Subcommittee, the Food and Drug Administration (even if drugs or medical devices are not involved), and other Federal Regulating Agencies. By signing this form, you authorize such inspection.

VACHS IRB APPROVED

10/21/2013

Subject Name: ___________________________________ **Date:** ____________________

Title of Study: ___Pharmacogenetic-driven Warfarin Dosing Algorithm in Puerto Ricans_________

Principal Investigator: Giselle Rivera, Juan F. Feliu, Jorge Duconge

VA Caribbean Healthcare System
10 Casia Street
San Juan, PR 00921-3201

Sponsor: NIH

11. Withdrawal or refusal to participate will not result in loss of benefits:
Your participation in this research is voluntary. You may refuse to participate, or withdraw your consent and discontinue participation in the research at any time. Once the samples have been de-identified we may not be able to discard the sample you donated, because we will not be able to identify it. You may do so without penalty, or loss of benefits to which you are otherwise entitled. Your decision whether to participate will not affect your future medical care at VACHS.

Your physician may choose to terminate your participation in this study if in his judgment it is not of benefit to you or it is not indicated. If this is the case, you will be offered the best treatment available for your condition.

12. Explanation of whom to contact
Should any problem or question arise before or during the study with regards to this research, or with regards to any research related injury, you understand that you should contact Dr. Giselle Rivera and/or Dr. Juan F Feliu at (787)641-7582 ext. 31186, 11472.

13. Disclosure of significant new findings

You will be notified if information from other studies becomes available which could cause you to reconsider your participation in this study.

14. Approval by the human studies subcommittee

You understand that this study was approved by the Human Studies Subcommittee of this Institution.

15. Statement of voluntary participation

I voluntarily agree to participate in this study and in signing this form express my understanding of the procedures to be carried out and my willingness to participate. My agreement is based on my judgment, independent reasoning and freedom from coercion or undue influence. I will receive a signed and dated copy of this consent form.

16. Research subjects' rights: I have read or have had read to me all of the above. Dr. Giselle Rivera or her designee has explained the study to me and answered all of my questions. I have been told of the risks or discomforts and possible benefits of the study. I have been told of other choices of treatment available to me.

I understand that I do not have to take part in this study, and my refusal to participate will involve no penalty or loss of rights to which I am entitled. I may withdraw from this study at any time without penalty or loss of VACHS or other benefits to which I am entitled.

VACHS IRB APPROVED

10/21/2013

Subject Name: ________________________________ **Date:** ___________________

Title of Study: ___Pharmacogenetic-driven Warfarin Dosing Algorithm in Puerto Ricans_________

Principal Investigator: Giselle Rivera, Juan F. Feliu, Jorge Duconge

VA Caribbean Healthcare System
10 Casia Street
San Juan, PR 00921-3201

Sponsor: NIH

The results of this study may be published, but my records will not be revealed unless required by law.

In case there are medical problems, questions, concerns or complaints, I have been told I can call Dr. Giselle Rivera or Dr. Juan F Feliu at (787) 641-7582 Ext. 31186, 11472 during the day. After regular work hours, you may contact the physician on duty at the Emergency Room at the VACHS at (787) 641-7582.

If any medical problems related to this study occur the VACHS will provide emergency care.

In case of any question regarding your rights as patient in an investigation, call Dr. Jorge L. Martínez Díaz (Chairperson of the Human Studies Subcommittee of the IRB) at (787) 641-7582 extension 10485.

If I have any concerns or complaints about this study or the research team; or if I wish to talk to someone who is not part of the research team, I may contact Dr. Jorge L. Martínez Díaz (Chairperson of the Human Studies Subcommittee of the IRB) at (787) 641-7582 extension 10485. All calls will be confidential and if I choose, anonymous.

I voluntarily consent to participate in this study. I will receive a signed and dated copy of this consent form.

______________________	______________________	____________
Subject's Name (Printed)	*Subject's Signature*	*Date*
______________________	______________________	____________
Person *Obtaining Consent (Printed)*	*Signature of Person Obtaining Consent*	*Date*

VACHS IRB APPROVED

10/21/2013
