# Supplementary material for: A Novel Admixture-Based Pharmacogenetic Approach to Refine Warfarin Dosing in Caribbean Hispanics
Source: PLoS One. 2016 Jan 8;11(1):e0145480. doi: 10.1371/journal.pone.0145480 (PMC4706412; doi:10.1371/journal.pone.0145480)
Supplement: S3 Text — (PDF) [file pone.0145480.s003.pdf]

AUTHORIZATION FOR THE USE AND/OR RELEASE OF PROTECTED HEALTH INFORMATION FOR RESEARCH

A) Introduction: I have the right to decide who may review or use my Protected Health Information ("PHI") and/or my individually identifiable information (III), during the process of this research study. The type of information that may be used is described below. When I consider taking part in a research study, I must give permission for my PHI and/or III to be released from my doctors, clinics, and hospitals to the research team, for the specific purpose of this research study.

B)<u>What does this authorization relate to?</u> This authorization relates to the following research study:
<u>Pharmacogenetic-driven Warfarin Dosing Algorithm in Puerto Ricans</u>

PRINCIPAL INVESTIGATOR (in charge of the Research Team):	***IRB PROTOCOL NUMBER:***
GISELLE RIVERA-MIRANDA, Pharm.D.	00558
NAME OF RESEARCH SUBJECT:	***NAME OF SPONSOR:***
	N/A

C)<u>What information will be used and/or disclosed for research purposes?</u> The PHI that will be used and/or disclosed for research purposes may include some or all of my health records. The purpose of this information will be to achieve the intent identified in the Informed Consent or at the request of the individual and/or organization. This includes, but is not limited to: (Mark with an *x*)

X	Information provided by me directly to the Research Team		Any other medical or dental records needed by the Research Team
X	History and Physical Examination		HIV (testing or infections) records
	Discharge Summaries		Sickle Cell Anemia
X	Diagnostic/Laboratory test results		Mental Health (not psychotherapy notes)
	Immunizations		Photographs, videotapes, recordings, other images
	Allergy Reports		Billing Records
X	Prescriptions		Alcoholism or Alcohol Use
X	Consultations		Drug Abuse Information
X	Clinic and Progress Notes		Other: ______________________
	X Ray Films and reports		Other: ______________________

D) <u>Who will be allowed to use and/or release this information?</u>

I authorize the following persons, groups or organizations from the **VA Caribbean Healthcare System Research Program** to use and/or release the information described in this Release of Information/Authorization for the above referenced research study: (Mark with an *x*)

- ☐ **Treating providers**
- ☐ **Hospitals, clinics or other places where I have received treatment (other than VA)**
- ☐ **Other:**______________________
- ☒ **The Principal Investigator and the Research Staff**

E) <u>Who can access my PHI for the study?</u> The people and entities listed above may share my PHI (or the PHI of the individual(s) whom I have the authority to represent), with the following persons or groups for the research study: the Research Team, Institutional Review Board, Research Sponsor and its representatives, Research Organizations, the Department of Health & Human Services or other US or foreign government agencies as required by law, and to the Food and Drug Administration (FDA) or a person subject to the jurisdiction of the FDA in order to audit or monitor the quality, safety or effectiveness of the product or activity.

The **Research Team** includes the Principal Investigator, his/her staff, research coordinators, research technicians and other staff members who provide assistance to the Research Team. If there is a **Research Sponsor(s),** this shall include: ________N/A__________and any **Research Organizations** (monitors and auditors) who provided assistance to the **Research Sponsor(s)** including, but not limited to: __________N/A_____________.

Expiration date of this Authorization: This authorization is valid until the following date or event: (Mark with an *x*)

☐ Specify Date ___/___/____ ☒ End of the Study

☐ Indefinitely/None ☐ As authorized by the sponsor to destroy study documents

☐Other: ____________________

I have been informed that efforts will be made by the **VA Caribbean Healthcare System Research Program** to ensure that my PHI will not be shared with other people outside of the research study. I also have been informed that the PHI and/or III disclosed pursuant to this authorization may no longer be protected after its disclosure by Federal laws or regulations and may be subject to re-disclosure by the recipient.

F) I have the right:

1. To refuse to sign this form. Not signing the form will not affect my regular health care including treatment, payment, or enrollment in a health plan or eligibility for health care benefits. However, not signing the form will prevent me from participating in the research study above.
2. To review and obtain a copy of my personal health information collected during the study. However, it may be important to the success and integrity of the study that persons who participate in the study not be given access until the study is complete. The Principal Investigator has discretion to refuse to grant access to this information if it will affect the integrity of the study data during the course of the study. Therefore, my request for information may be delayed until the study is complete.
3. To cancel/revoke this release of information/authorization at any time. If I choose to cancel this release of information/authorization, I must notify the Principal Investigator for this study **in writing** at: GISELLE RIVERA, PHARM.D.__(***VA Caribbean Healthcare System, Research Service 151, #10 Casia St., San Juan Puerto Rico 00921)***. However, even if I cancel this release of information/authorization, the Research Team, Research Sponsor(s) and/or the Research Organizations may still use information about me that was collected as part of the research project between the date I signed the current form and the date I cancel the authorization. This is to protect the quality of the research results. I understand that canceling this authorization may end my participation in this study.
4. To receive a copy of this form.

I have had the opportunity to review and ask questions regarding this release of information/authorization form. By signing this release of information/authorization, I am confirming that it reflects my wishes.

____ ______________________

Printed name of Individual/Legal Representative ***Social Security Number***

____ ______________________

Signature of Individual/Legal Representative ***Date***

**If signed by a legal representative; state the relationship and identify below the authority to act on behalf of the individual's behalf.*

***Individual is:** ☐ Incompetent ☐ Other: ________________

***Legal Authority:** ☐ Power of Attorney Healthcare ☐ Legally Authorized Representative, write the relationship;_________________________
